# Prevalence and Molecular Characteristics of Extended-Spectrum and AmpC β-Lactamase Producing *Escherichia coli* in Grazing Beef Cattle

**DOI:** 10.3389/fmicb.2019.03076

**Published:** 2020-01-09

**Authors:** Shinyoung Lee, Lin Teng, Nicolas DiLorenzo, Thomas A. Weppelmann, Kwangcheol Casey Jeong

**Affiliations:** ^1^Department of Animal Sciences, Emerging Pathogens Institute, University of Florida, Gainesville, FL, United States; ^2^Department of Animal Sciences, Institute of Food and Agricultural Sciences, University of Florida, Gainesville, FL, United States; ^3^North Florida Research and Education Center, Institute of Food and Agricultural Sciences, University of Florida, Marianna, FL, United States; ^4^Herbert Wertheim College of Medicine, Florida International University, Miami, FL, United States

**Keywords:** *E. coli*, ESBL, AmpC, antibiotic resistance, grazing beef cattle

## Abstract

The emergence of extended-spectrum β-lactamase (ESBL) and AmpC β-lactamase producing *Escherichia coli* represent a contemporary public health threat. ESBL and AmpC β-lactamase genes translocate between chromosomes and plasmids, facilitating rapid spread throughout the environment. In this study, ESBL/AmpC producing bacteria were isolated from beef cattle farms with seldom antibiotic use. Eleven farms out of 17 tested, had ESBL/AmpC producing *E. coli* in animals, soil, and forage samples. Fifty-nine CTX-M or CMY-2 positive *E. coli* isolates were further characterized with whole-genome sequencing. The isolates commonly carried CMY-2, TEM, or CTX-M genes, and over half encoded both CTX-M and TEM genes. Using comparative genomics, antimicrobial resistance genes from 12 classes of antimicrobial were identified and confirmed by antibiotic susceptibility test, revealing multidrug resistance against multiple classes of antibiotics. Virulence factors related to adherence, invasion, iron uptake, and bacterial secretion systems were shared by all isolates; some of which were identified as enteropathogenic *E. coli*. Phylogenetic analyses revealed a pattern of close genetic relatedness, suggesting that ESBL/AmpC producing *E. coli* were transmitted among farms as well as independent evolution within farms. Our results indicate that ESBL and AmpC β-lactamases prevail in food animal production system regardless antibiotic use and have the characteristics for zoonotic transmission.

## Introduction

Extended-spectrum β-lactamase (ESBL) and AmpC β-lactamase have become prevalent in *Enterobacteriaceae*, representing a contemporary public health threat (Rawat and Nair, 2010). ESBL or overexpressed AmpC β-lactamases allow bacteria such as *Escherichia coli* to survive from treatment with most broad-spectrum β-lactams, thereby limiting their efficacy in medicine (Horton et al., 2011; Doi et al., 2013). ESBL/AmpC producing *E. coli* were mainly associated with hospital infections, and those β-lactamase genes were originally located in chromosomal DNA of *Kluyvera* spp. and *Citrobacter* spp., respectively (Jacoby, 2009; Verdet et al., 2009). Due to the increased use of β-lactams and subsequent relocation of ESBL and AmpC β-lactamase genes to plasmids, ESBL/AmpC producing *E. coli* are widely disseminated into the environment and into the food-producing animals (Blaak et al., 2014; Ma et al., 2018, 2019). This allows for zoonotic transmission to humans through contaminated food products, creating a feedback loop for evolution and positive selection of new resistance genes (Ewers et al., 2012; Ibrahim et al., 2016). Thus, the emergence of β-lactam resistant bacteria in food-producing animals represents both a challenge to global health and a potential critical control point (Doi et al., 2013; Petty et al., 2014).

Although the awareness of ESBL/AmpC producing bacteria in food animals has increased, much work has focused on confined animal feeding operations with extensive antibiotic supplementation in feedstocks (Cottell et al., 2013). More recently, a high prevalence of cefotaxime resistant bacteria (CRB) was identified in beef cattle on cow/calf operations, where the grazing animals were not supplemented with antibiotics except medical necessity to treat sporadic infections (Markland et al., 2019). Additionally, soil samples from the environment contained high concentrations of CRB, raising a question whether the soil was merely contaminated by feces shed by the cattle, or it was the origin of the CRB identified in the cattle. Given the multiple evidences of existing natural antibiotic resistomes (Wellington et al., 2013), we hypothesized that ESBL/AmpC producing *E. coli* isolates in commercial beef farms were acquired through outside sources and spread among farms, as well as independent evolution in farms. To investigate the origins of multi-drug resistant bacteria isolated from food animals raised without antibiotic supplementation as growth promoters, a sample of ESBL/AmpC producing *E. coli* were further characterized via comparative genomics with whole genome sequencing. Additionally, phenotypic characteristics were studied with antibiotic susceptibility testing and functional genomics, to evaluate mechanisms of antimicrobial resistance and the potential for zoonotic transmission from animals to humans and between other bacteria via plasmid exchange.

## Materials and Methods

### Isolation of CTX-M/CMY-2 Producing Bacteria and *E. coli*

**FIGURE 1:**
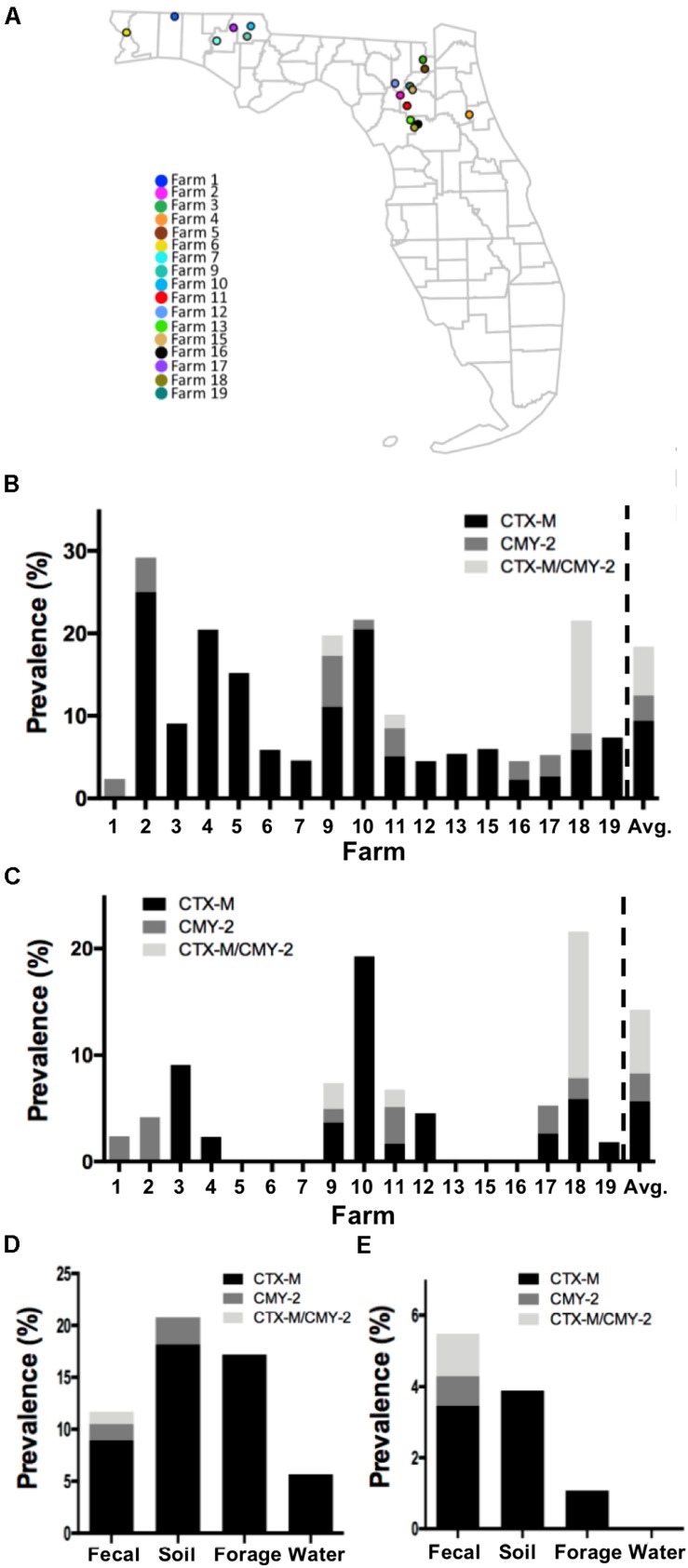
Prevalence of CTX-M/CMY-2 producing isolates. **(A)** All the samples used in this study were collected from 17 commercial farms located in Northern Florida. The prevalence of CTX-M/CMY-2 producing bacteria **(B)** and CTX-M/CMY-2 producing *E. coli* **(C)** were presented by different farms. Depending on the sample type, the prevalence of CTX-M/CMY-2 producing bacteria **(D)** and CTX-M/CMY-2 producing *E. coli* **(E)** were also compared.

Samples (*n* = 1,098) including animal feces (*n* = 840) and environmental samples (forage: 93, soil: 77, and water: 88) were collected from 17 commercial beef cattle farms in Florida (Figure 1A) between February and June 2016 and plated on MacConkey agar containing cefotaxime (4 μg/mL). CRB colonies from each sample were screened for the CTX-M and CMY-2 genes using a polymerase chain reaction (PCR) assay as previously described (Edelstein et al., 2003). If a sample contained less than 10 colonies, we screened all the colonies. If CRB colonies were more than 10 colonies per sample, we randomly selected ten CRB colonies with different morphologies for the screening. The PCR conditions were as follows: 94°C for 5 min, 35 cycles at 94°C for 30 s, 52°C for 30 s, 72°C for 1 min and 72°C for 10 min for a final extension. The primer sets used to screen the CTX-M and CMY-2 genes: KCP685 (5′-TTTGCGATGTGCAGTACCAGTAA-3′) and KCP686 (5′-CGATATCGTTGGTGGTGCCATA-3′) for the CTX-M gene and KCP556 (5′-ATGATGAAAAAATCGTTATGC-3′) and KCP557 (5′-TTGCAGCTTTTCAAGAATGCGC-3′) for the CMY-2 gene (Mir et al., 2016). *E. coli* were selected on CHROMagar *E. coli* (CHROMagar, France) after overnight incubation at 37°C. The concentration of CTX-M or CMY-2 encoding CRB in each sample was calculated by multiplying the percentage of CTX-M/CMY-2 encoding CRB in 10 isolates with the concentration of CRB in the sample.

### Whole Genome Sequencing and Phylogeny Analysis

Genomic DNA of 59 ESBL/AmpC producing *E. coli* isolates were extracted with the DNeasy blood and tissue kit (Qiagen, United States) prior to DNA library construction with Nextera XT sample preparation kit (Illumina, United States). Strains were sequenced using an Illumina MiSeq with cartridges providing 2 × 250 paired-end read coverage. Sequencing reads were trimmed using Sickle (Joshi and Fass, 2011) followed by *de novo* assembly with SPAdes 3.0 (Bankevich et al., 2012). All of the assembled contigs from 59 isolates were reordered based on the whole genome sequences of Sakai (NC_002695.1) as the reference using Mauve (Rissman et al., 2009). Parsnp software of Harvest suite (v1.2) (Treangen et al., 2014) was applied to generate maximum likelihood phylogenetic trees based on core-genome single nucleotide polymorphism (SNP). Recombination within assembled sequences was detected with PhiPack (Bruen et al., 2006), and the phylogenetic tree was generated with FastTree (Price et al., 2010) using 1,000 bootstrap replicates embedded in Parsnp software. Final tree annotations were modified using FigTree^1^. All of the reference genomes were downloaded from Enterobase^2^, and Parsnp program was also used to generate the phylogenetic trees based on the sequencing type (ST).

### Minimum Inhibitory Concentration and Antimicrobial Susceptibility Test

The antibiotic resistance of 59 ESBL/AmpC producing *E. coli* isolates was evaluated by minimum inhibitory concentration (MIC) of cefotaxime and antimicrobial susceptibility test (AST). MIC testing was performed using a micro-broth dilution method that followed the Clinical and Laboratory Standards Institute (CLSI) guidelines (CLSI, 2015, 2016). The concentration of cefotaxime was serially diluted with Mueller Hinton Broth (MHB) from 0 to 256 μg/mL with the MIC defined by the highest concentration among three replicates. The ESBL-producing strain KCJ1409 was used as a positive control, and DH5α as a negative control (Mir et al., 2016). The standard Kirby Bauer disk diffusion method on Mueller Hinton Agar (MHA) was used to test susceptibility against the following 13 antibiotics: amikacin (30 μg), ampicillin (10 μg), amoxicillin/clavulanic acid (30 μg), sulfisoxazole (250 μg), ceftiofur (30 μg), chloramphenicol (30 μg), cephalothin (30 μg), gentamicin (10 μg), nalidixic acid (30 μg), streptomycin (10 μg), sulfamethoxazole/trimethoprim (23.75 μg/1.25 μg), tetracycline (30 μg), and colistin (10 μg) (BD, United States). *E. coli* (ATCC 35401), *Staphylococcus aureus* (ATCC 25923), and *Pseudomonas aeruginosa* (ATCC 27853) were used as controls.

### Genetic Characterization of ESBL/AmpC Producing *E. coli*

The Center for Genomic Epidemiology (CGE) database was used to identify bacterial species, β-lactamase genes, multi locus sequence types (MLST), serotypes, plasmid replicon types, and plasmid MLST (pMLST) of each isolate with the *de novo* assembled contigs (Zankari et al., 2012). Phylogenetic groups were generated *in silico* based on the presence of *chuA*, *yjaA*, and *TSPE4.C2* genes as previously described (Clermont et al., 2000). The Resistance Gene Identifier (RGI) in Comprehensive Antibiotic Resistance Database (CARD) (version 1.2.1) was used to create antimicrobial resistance gene (ARG) profiles for each isolate based on homology and SNP models (Jia et al., 2017). To investigate virulence genes, whole genome sequence of the isolates was compared to the reference sequences of virulence genes in Virulence Factors Database (VFDB) (last update: October 2017) by BLAST (Chen et al., 2016). Only genes with query and subject coverage higher than 50%, were considered present. To investigate whether CTX-M and CMY-2 genes were located in plasmid or chromosome, the contigs containing the CTX-M and CMY-2 genes were blasted to the NCBI database using BLASTn, and the location of the CTX-M and CMY-2 genes were determined along with any insertion sequences (IS). The genetic environments of CTX-M and CMY-2 genes were compared with GenBank files of sequenced strains and visualized by the Easyfig tool (Sullivan et al., 2011).

### Comparison of Genome Function

To investigate the functional genomic differences between the isolates, the functional modules of the isolates were reconstructed and compared using Kyoto Encyclopedia of Genes and Genomes (KEGG) (Kanehisa et al., 2016a, b). The annotated amino acid sequences were submitted to BlastKOALA (Kanehisa et al., 2016b) to receive corresponding KEGG Orthology (KO) numbers of the coding sequences (CDS) prior to reconstruction of the functional modules using KO numbers of each isolate. Orthologous proteins were identified and compared using InParanoid standalone version 4.1 program as previously described (Sonnhammer and Ostlund, 2015). Translated CDS of each isolate were downloaded from NCBI and used as the input file. The output files of InParanoid program were further processed with in-house developed Python scripts to distinguish between core and unique proteins.

### Epithelial Cell Adherence Assay

The ability of the ESBL/AmpC producing *E. coli* to adhere to human intestinal epithelial cells evaluated via the following protocol. Caco-2 cells were maintained in Dulbecco’s modified Eagle medium (DMEM; Corning Inc., United States) supplemented with 20% fetal bovine serum (FBS; Hyclone, United States) at 37°C and 5% CO_2_. Caco-2 cells were seeded on a 24-well polystyrene plate with approximately 10^5^ cells/well and grown to 90% confluence. Overnight culture of bacteria was seeded to new LB media (1:250) and cultured at 37°C for 8 h with shaking. The bacterial cells washed three times with sterile phosphate buffered saline (PBS). Approximately 10^6^ bacterial cells were resuspended in 500 μL DMEM, inoculated into the wells containing Caco-2 cells (a multiplicity of infection; MOI of 10), and incubated for 3 h. After first incubation, all the wells were replaced by fresh DMEM followed by another incubation for 3 h. After 6 h incubation in total, the media was removed from each well, and Caco-2 cells washed three times with sterile PBS to eliminate unattached bacterial cells. Caco-2 cells were lysed and adhered bacterial cells were detached by adding 1 mL of 1% Triton X-100 and pipetting. Serial dilutions of the suspensions were spread on LB and colonies on the agar plates were enumerated after overnight incubation at 37°C. Each experiment was conducted twice in duplicate with EDL933 and DH5α as positive and negative controls, respectively. Statistical differences were analyzed by the one-way analysis of variance (ANOVA) followed by Tukey’s test (α = 0.05).

## Results

### Prevalence and Concentration of ESBL and AmpC Producing *E. coli*

Of the 17 commercial farms (Figure 1A), 16 farms contained CTX-M positive bacteria with the prevalence ranging from 2.3 to 25.0% (average = 9.44%; Figure 1B). CMY-2 positive bacteria were identified from eight farms with the prevalence ranging from 1.2 to 6.17% (average = 3.02%; Figure 1B), and three farms had bacteria with both CTX-M and CMY-2 genes (Figure 1B). The concentration of CTX-M and CMY-2 positive bacteria were variable among farms with an average of 1.53 log CFU/g of feces (Table 1). Approximately 53% (9/17) of farms had CTX-M positive *E. coli*, 35% of the farms (6/17) contained CMY-2 positive *E. coli*, and 18% of the farms (3/17) carried *E. coli* with both CTX-M and CMY-2 genes (Figure 1C). The average prevalence of *E. coli* isolates with CTX-M, CMY-2, or both genes on farms was 5.66, 2.62, and 5.96%, respectively (Figure 1C). The average concentration of CTX-M or CMY-2 encoding *E. coli* among the farms was 1.49 and 1.51 log CFU/g of feces, respectively (Table 1).

**TABLE 1:** Concentration of CTX-M and CMY-2 positive bacteria and *E. coli* by farm.

**Farm**	**CTX-M positive**				**CMY-2 positive**			
	**Bacteria**		***E. coli***		**Bacteria**		***E. coli***	
	**Concentration (LogCFU/g)**	**SD^b^**	**Concentration (LogCFU/g)**	**SD**	**Concentration (LogCFU/g)**	**SD**	**Concentration (LogCFU/g)**	**SD**
1	–^a^	–	–	–	1.10	–	1.10	–
2	1.59	0.31	–	–	1.80	–	1.80	–
3	2.57	0.61	2.33	0.34	–	–	–	–
4	1.51	0.42	1.30	–	–	–	–	–
5	1.68	0.78	–	–	–	–	–	–
6	1.14	–	–	–	–	–	–	–
7	1.38	0.34	–	–	–	–	–	–
9	1.46	0.40	1.49	0.42	1.17	0.17	1.31	0.19
10	1.70	0.63	1.61	0.53	3.21	–	–	–
11	1.29	0.24	1.49	0.12	1.30	0.17	1.30	0.17
12	1.10	–	1.01	–	–	–	–	–
13	1.22	0.24	–	–	–	–	–	–
15	1.29	0.34	–	–	–	–	–	–
16	1.10	–	–	–	1.10	–	–	–
17	1.18	–	1.18	–	1.80	–	1.80	–
18	1.63	1.07	1.63	1.07	1.76	1.14	1.76	1.14
19	1.17	0.12	1.35	–	–	–	–	–
Average	1.44	0.36	1.49	0.37	1.66	0.70	1.51	0.31

*^*a*^The number of positive sample is zero or one. ^*b*^SD, standard deviation.*

As potential routes, antibiotic resistant bacteria can be transferred to animals through the environment, without the exposure of antibiotics before (Mir et al., 2016, 2018; Teng et al., 2019). To understand whether farm environment could be outside sources of antibiotic resistant bacteria in animals, and to identify which environmental factors are mostly critical to the animals, the prevalence and concentration of CTX-M and CMY-2 positive isolates were compared based on the sample types. The prevalence of CTX-M producing bacteria in fecal, soil, forage, and water samples were 8.9, 18.1, 17.2, and 5.6%, respectively (Figure 1D). CMY-2 producing bacteria were found in 1.5 and 2.5% of fecal and soil samples, respectively (Figure 1D). The prevalence of CTX-M and CMY-2 producing bacteria were higher in soil (20.78%) and forage samples (17.2%) than fecal samples (11.7%), and only fecal samples contained bacteria with both CTX-M and CMY-2 genes (1.19%) (Figure 1D). In contrast, the prevalence of CTX-M or CMY-2 producing *E. coli* in cattle on farms positive with these bacteria was higher than environmental samples, where the prevalence was 5.49% in feces, 3.89% in soil, and 1.07% in forage (Figure 1E). The concentrations of CTX-M/CMY-2 producing bacteria and *E. coli* were higher in environmental samples compared to animal samples (Table 2).

**TABLE 2:** Concentration of CTX-M and CMY-2 positive bacteria and *E. coli* by sample type.

	**CTX-M positive**					
	**Bacteria**			***E. coli***		
**Sample type**	**Positive samples (*n*)**	**Concentration (logCFU/g or L)**	**SD^b^**	**Positive samples (*n*)**	**Concentration (logCFU/g or L)**	**SD**
Fecal	85	1.53	0.60	39	1.59	0.67
Forage	16	4.90	1.54	1	2.27	–^a^
Soil	14	4.31	0.78	3	4.78	0.40
Water	5	–1.01	0.51	0	–	–

	**CMY-2 positive**					
	**Bacteria**			***E. coli***		
**Sample type**	**Positive samples (*n*)**	**Concentration (logCFU/g or L)**	**SD**	**Positive samples (*n*)**	**Concentration (logCFU/g or L)**	**SD**

Fecal	23	1.52	0.80	17	1.56	0.80
Forage	0	–	–	0	–	–
Soil	2	4.04	0.37	0	–	–
Water	0	–	–	0	–	–

*^*a*^The number of positive sample is zero or one. ^*b*^SD, standard deviation.*

### Genetic Relationship Within Commercial Farms Isolates

Genetic relatedness among commercial farm isolates was investigated to understand the transmission of ESBL/AmpC producing *E. coli* among commercial farms, including 56 strains from feces (calf: 45 and cow: 11), and three environmental strains (one from forage and two from soil). Seventeen sequencing types were shown in our isolates, and the predominant MLST was ST10 (*n* = 11) (Table 3). As the other sequencing types, there are ST5727 (*n* = 10), ST1266 (*n* = 1), ST4086 (*n* = 4), ST906 (*n* = 2), ST744 (*n* = 8), ST4086 (*n* = 2), ST6353 (*n* = 1), ST685 (*n* = 1), ST101 (*n* = 1), ST6416 (*n* = 2), ST1121 (*n* = 2), ST1674 (*n* = 1), ST2509 (*n* = 1), ST1172 (*n* = 6), ST6465 (*n* = 3), ST6466 (*n* = 1), and ST20 (*n* = 2) (Table 3). Most of the isolates had same sequencing types if the strains were isolated from the same farm with a few exceptions. Forty-four percent (26/59) of the isolates were allocated to phylogroup A, 34% (20/59) to phylogroup B1, 18% (11/59) to phylogroup B2, and only two isolates were belonged to phylogroup D (Table 3).

**TABLE 3:** Characterization of ESBL/AmpC producing *E. coli*.

**Strain**	**Species**	**Source (Farm#/Source)**	**β-lactamase genes**	**MLST**	**Serotype**	**Phylo-groups**	**Location of β-lactamase**	**Predicted plasmids**	**pMLST**
KCJK9^a^	*E. coli*	1/Calf feces	CMY-2	1266	O161:H34	B2	Plasmid	IncX, IncP	NA^b^
KCJK326^a^	*E. coli*	2/Calf feces	CMY-2	4086	:H10	B1	Plasmid	Col156, IncA/C2, ColRNAI	NA
KCJK327	*E. coli*	2/Calf feces	CMY-2	4086	:H10	B1	Plasmid	Col156, IncA/C2, ColRNAI	NA
KCJK328	*E. coli*	2/Calf feces	CMY-2	4086	:H10	B1	Plasmid	Col156, IncA/C2, ColRNAI	NA
KCJK329	*E. coli*	2/Calf feces	CMY-2	4086	:H10	B1	Plasmid	Col156, IncA/C2, ColRNAI	NA
KCJK330	*E. coli*	2/Calf feces	CMY-2	4086	:H10	B1	Plasmid	Col156, IncA/C2, ColRNAI	NA
KCJK467^a^	*E. coli*	2/Forage	CTX-M-1, TEM-1A	5727	O27:H9	B2	Plasmid	IncY, IncR, ColRNAI	NA
KCJK500	*E. coli*	3/Calf feces	CTX-M-1, TEM-1A	5727	O27:H9	B2	Plasmid	IncY, IncR, ColRNAI	NA
KCJK501^a^	*E. coli*	3/Calf feces	CTX-M-1, TEM-1A	5727	O27:H9	B2	Plasmid	IncY, IncR, ColRNAI	NA
KCJK502	*E. coli*	3/Calf feces	CTX-M-1, TEM-1A	5727	O27:H9	B2	Plasmid	IncY, IncR, ColRNAI	NA
KCJK506	*E. coli*	3/Calf feces	CTX-M-1, TEM-1A	5727	O27:H9	B2	Plasmid	IncY, IncR, ColRNAI	NA
KCJK507^a^	*E. coli*	3/Calf feces	CTX-M-1, TEM-1A	5727	O27:H9	B2	Plasmid	IncY, IncR, ColRNAI	NA
KCJK511	*E. coli*	3/Calf feces	CTX-M-1, TEM-1A	5727	O27:H9	B2	Plasmid	IncY, IncR, ColRNAI	NA
KCJK512	*E. coli*	3/Calf feces	CTX-M-1, TEM-1A	5727	O27:H9	B2	Plasmid	IncY, IncR, ColRNAI	NA
KCJK513	*E. coli*	3/Calf feces	CTX-M-1, TEM-1A	5727	O27:H9	B2	Plasmid	IncY, IncR, ColRNAI	NA
KCJK514^a^	*E. coli*	3/Calf feces	CTX-M-1, TEM-1A	5727	O27:H9	B2	Plasmid	IncY, IncR, ColRNAI	NA
KCJK2721^a^	*E. coli*	4/Calf feces	CTX-M-1	906	O150:H8	B1	Plasmid	IncF, IncHI, IncQ, ColRNAI	F-:A8:B24, HI1:ST-2
KCJK2722^a^	*E. coli*	4/Calf feces	CTX-M-1	906	O150:H8	B1	Plasmid	IncF, IncHI, IncQ, ColRNAI	F-:A8:B24, HI1:ST-2
KCJK3864^a^	*E. coli*	9/Calf feces	CTX-M-1, TEM-1A	744	O89:H9	A	Plasmid	IncF, IncR, IncQ, ColRNAI, ColMG828	F14:A6:B45
KCJK3886^a^	*E. coli*	9/Calf feces	CTX-M-1, TEM-1A	744	O89:H9	A	Plasmid	IncF, IncR, IncQ, ColRNAI, ColMG828	F14:A6:B45
KCJK3889	*E. coli*	9/Calf feces	CTX-M-1, TEM-1A	744	O89:H9	A	Plasmid	IncF, IncR, IncQ, ColRNAI, ColMG828	F14:A6:B45
KCJK3893	*E. coli*	9/Calf feces	CTX-M-1, TEM-1A	744	O89:H9	A	Plasmid	IncF, IncR, IncQ, ColRNAI, ColMG828	F14:A6:B45
KCJK3909^a^	*E. coli*	9/Calf feces	CTX-M-1, TEM-1A	744	O89:H9	A	Plasmid	IncF, IncR, IncQ, ColRNAI, ColMG828	F14:A6:B45
KCJK3910	*E. coli*	9/Calf feces	CTX-M-1, TEM-1A	744	O89:H9	A	Plasmid	IncF, IncR, IncQ, ColRNAI, ColMG828	F14:A6:B45
KCJK3915^a^	*E. coli*	9/Calf feces	CMY-2	4086	O53:H10	B1	Plasmid	IncF, IncA/C, ColMG828	C1:A-:B16
KCJK3920	*E. coli*	9/Calf feces	CTX-M-1, TEM-1A	744	O89:H9	A	Plasmid	IncF, IncR, IncQ1, ColRNAI, ColMG828	F14:A6:B45
KCJK3945^a^	*E. coli*	9/Calf feces	CMY-2	6353	O132:H18	B1	Plasmid	IncHI, IncF, ColRNAI, IncB/O/K/Z	F6:A-:B, HI1:Unknown
KCJK4127^a^	*E. coli*	10/Cow feces	CTX-M-27, TEM-1A	10	:H32	D	Plasmid	IncF, IncR	F2:A-:B-
KCJK4137	*E. coli*	10/Cow feces	CTX-M-27, TEM-1A	10	:H32	A	Plasmid	IncF, IncR	F2:A-:B-
KCJK4139	*E. coli*	10/Cow feces	CTX-M-27, TEM-1A	10	:H32	A	Plasmid	IncF, IncR, ColpVC	F2:A-:B-
KCJK4140^a^	*E. coli*	10/Cow feces	CTX-M-32, TEM-1A	685	O123:H32	A	Chromosome	IncX	NA
KCJK4144	*E. coli*	10/Cow feces	CTX-M-27, TEM-1A	10	:H32	A	Plasmid	IncF, IncR, ColRNAI	F2:A-:B-
KCJK4148	*E. coli*	10/Cow feces	CTX-M-27, TEM-1A	10	:H32	A	Plasmid	IncF, IncR	F2:A-:B-
KCJK4158	*E. coli*	10/Cow feces	CTX-M-27, TEM-1A	10	:H32	A	Plasmid	IncF, IncR	F2:A-:B-
KCJK4162^a^	*E. coli*	10/Cow feces	CTX-M-27, TEM-1A	10	:H32	A	Plasmid	IncF, IncR	F2:A-:B-
KCJK4166	*E. coli*	10/Cow feces	CTX-M-27, TEM-1A	10	:H32	A	Plasmid	IncF, IncR	F2:A-:B-
KCJK4176	*E. coli*	10/Cow feces	CTX-M-27, TEM-1A	10	:H32	A	Plasmid	IncF, IncR, ColpVC	F2:A-:B-
KCJK4181^a^	*E. coli*	10/Soil	CTX-M-27, TEM-1A	10	:H32	A	Plasmid	IncF, IncR	F2:A-:B-
KCJK4201	*E. coli*	10/Soil	CTX-M-27, TEM-1A	10	:H32	A	Plasmid	IncF, IncR	F2:A-:B-
KCJK4405^a^	*E. coli*	12/Cow feces	CTX-M-1	101	:H40	B1	Plasmid	IncF	F19:A-:B38
KCJK4726^a^	*E. coli*	11/Calf feces	CTX-M-1, TEM-1A	6416	O108:H29	B1	Plasmid	IncY, IncR	NA
KCJK4727^a^	*E. coli*	11/Calf feces	CMY-2	1121	:H48	A	Plasmid	IncF, IncA/C2	C1:A6:B16
KCJK4729^a^	*E. coli*	11/Calf feces	CTX-M-1, TEM-1A	6416	O108:H29	B1	Plasmid	IncY, IncR	NA
KCJK4744^a^	*E. coli*	11/Calf feces	CMY-2	1121	:H48	A	Plasmid	IncF, IncA/C2	C1:A6:B16
KCJK4934^a^	*E. coli*	17/Calf feces	CMY-2	1674	O11:H25	D	Plasmid	IncI1	I1:ST-2
KCJK4935^a^	*E. coli*	17/Calf feces	CMY-2	2509	:H23	A	Plasmid	IncA/C2	NA
KCJK4937^a^	*E. coli*	17/Calf feces	CTX-M-55	744	O89:H9	A	Plasmid	IncF, IncQ, ColRNAI	F18:A-:B1
KCJK5106^a^	*E. coli*	18/Calf feces	CMY-2	1172	O184:H48	B1	Plasmid	IncF, IncA/C2, ColRNAI	F16:A-:B23
KCJK5108^a^	*E. coli*	18/Calf feces	CMY-2	1172	O184:H48	B1	Plasmid	IncF, IncA/C2, ColRNAI	F16:A-:B23
KCJK5139	*E. coli*	18/Calf feces	CTX-M-32	6465	:H9	A	Chromosome	NA	NA
KCJK5142^a^	*E. coli*	18/Calf feces	CTX-M-32	6465	:H9	A	Chromosome	NA	NA
KCJK5143^a^	*E. coli*	18/Calf feces	CTX-M-15, TEM-1B	6466	O127:H36	A	Plasmid	IncF	F-:A-:B53
KCJK5144^a^	*E. coli*	18/Calf feces	CMY-2	20	:H2	B1	Plasmid	IncF, IncHI, IncA/C2	F2:A8:B12, HI1:Unknown
KCJK5146^a^	*E. coli*	18/Calf feces	CMY-2	20	O128ac:H2	B1	Plasmid	IncF, IncHI, IncA/C2	F2:A8:B12, HI1:ST-2
KCJK5148^a^	*E. coli*	18/Calf feces	CMY-2	1172	O184:H48	B1	Plasmid	IncF, IncA/C2, ColRNAI	F16:A-:B23
KCJK5158^a^	*E. coli*	18/Calf feces	CMY-2	1172	O184:H48	B1	Plasmid	IncF, IncA/C2, ColRNAI	F16:A-:B23
KCJK5161	*E. coli*	18/Calf feces	CMY-2	1172	O184:H48	B1	Plasmid	IncF, IncA/C2, ColRNAI	F16:A-:B23
KCJK5162	*E. coli*	18/Calf feces	CMY-2	1172	O184:H48	B1	Plasmid	IncF, IncA/C2, ColRNAI	F16:A-:B23
KCJK5395^a^	*E. coli*	19/Calf feces	CTX-M-32	6465	:H9	A	Chromosome	NA	NA

*^*a*^The isolates are representative isolates to further analyze. ^*b*^NA, not available.*

**FIGURE 2:**
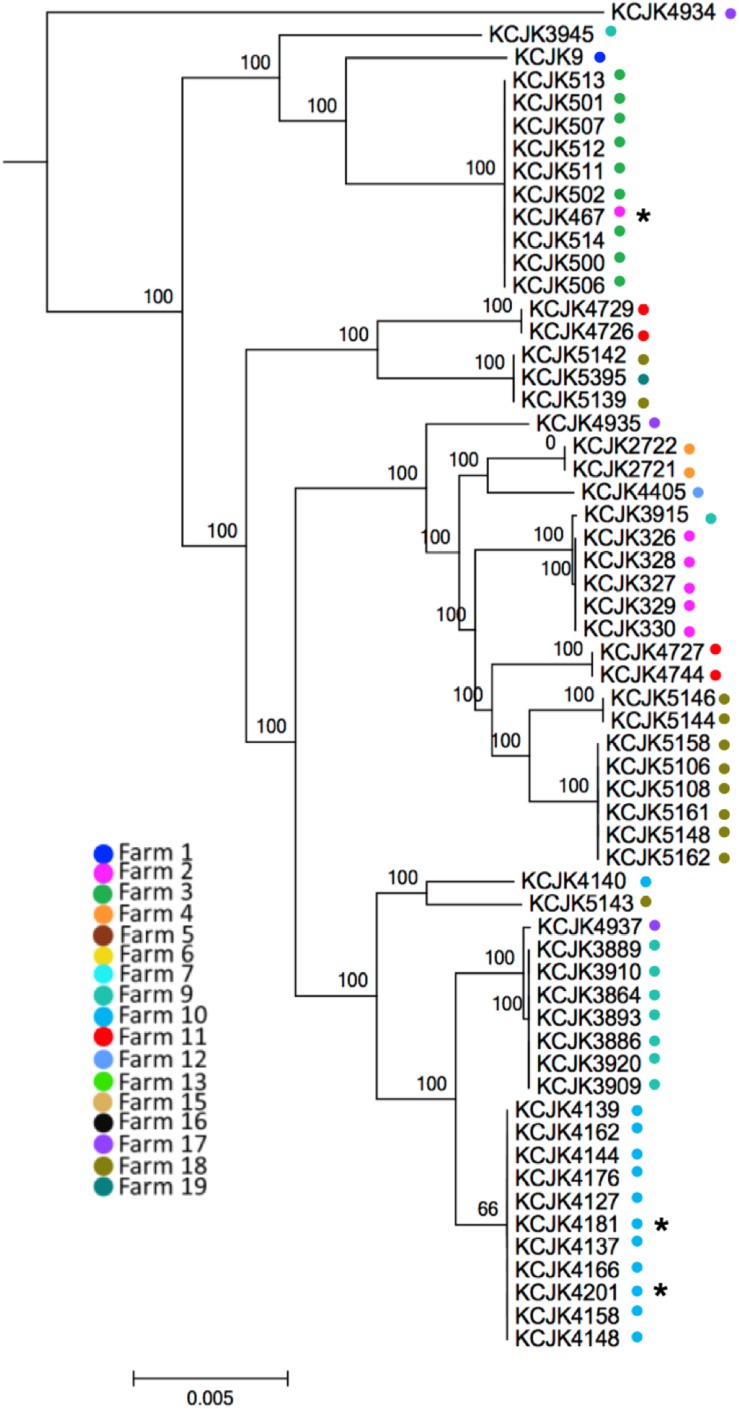
Phylogenetic tree between CTX-M/CMY-2 producing *E. coli* isolates based on core-genome alignment The core-genome phylogenetic tree was generated using Parsnp. The isolates from different farms were indicated by different colored dots. Environmental isolates were marked with an asterisk (^∗^). The scale bars indicate the mean numbers of nucleotide substitution per site.

Through the phylogeny analysis based on core-genome SNPs, fifty-nine CTX-M/CMY-2 positive *E. coli* isolates were grouped into 17 clades, consistent with their MLST (Figure 2 and Table 3). Most of the isolates were clustered specifically based on farm location (Figure 2), showing farm specificity. However, some clusters contained strains from different farms. For example, KCJK467, which was isolated from a forage sample in farm 2, was clustered with cattle isolates from farm 3. In addition, KCJK5139 and KCJK5142 were located in the same cluster with KCJK5395, even though they were isolated from different farms, farm 18 and 19, respectively (Figure 2). This suggests that there are carriers outside beef farms, which can cause the transmission of ESBL/AmpC producing *E. coli* between farms.

### Antimicrobial Susceptibility Test

**FIGURE 3:**
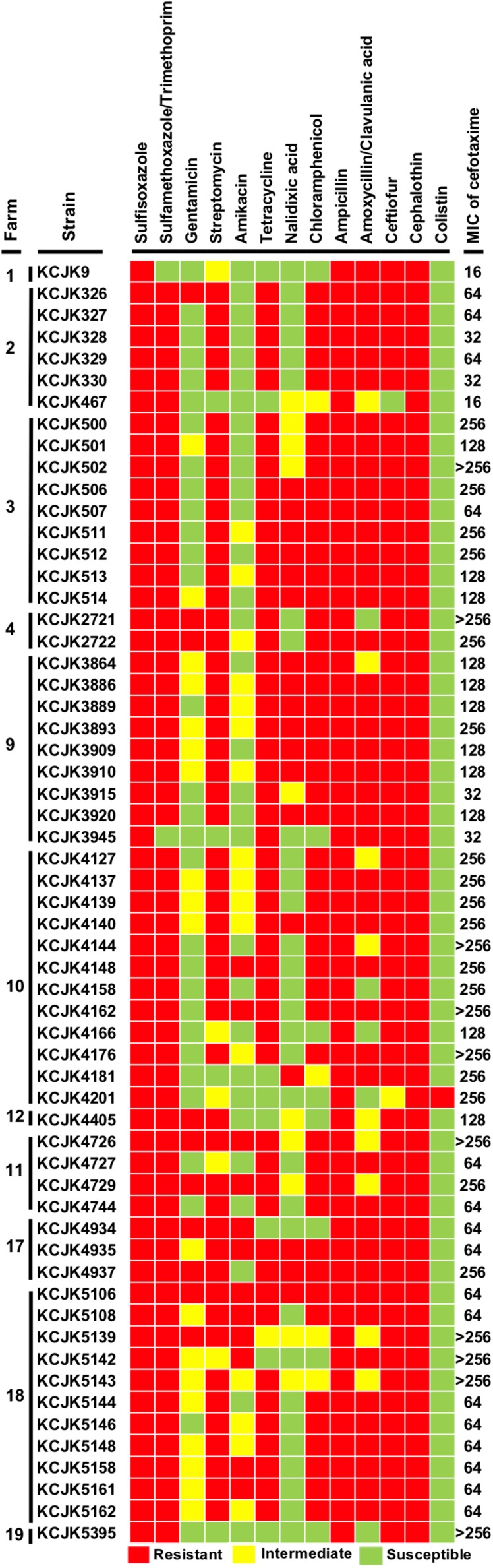
Antibiotic susceptibility test and MIC of cefotaxime All of the 59 CTX-M/CMY-2 producing *E. coli* isolates were tested antibiotic susceptibility with 13 antibiotics and MIC with cefotaxime. Antibiotic resistance ability is indicated by colored squares as follows: red, resistant; yellow, intermediate; and green, susceptible. The MIC data represents the highest concentration of triplicate.

All of the isolates showed a MIC of cefotaxime equal or greater than 16 μg/mL, and most of the isolates harboring the CTX-M gene had higher MIC than the isolates with CMY-2 gene (Figure 3 and Table 3). Ten isolates (16.4%) required more than 256 μg/mL of cefotaxime to be inhibited their growth. Antibiotic susceptibility patterns with 13 different antibiotics revealed multi-drug resistance against 4 to 12 different antibiotics. All tested strains were resistant to sulfisoxazole, ampicillin, and cephalothin. Apart from gentamicin, nalidixic acid, amikacin, amoxicillin/clavulanic acid, and colistin, more than 70% of the isolates showed antibiotic resistance against other 8 antibiotics (sulfisoxazole: 100%, tetracycline: 84%, sulfamethoxazole/trimethoprim: 96%, chloramphenicol: 79%, streptomycin: 84%, ampicillin: 100%, cephalothin: 100%, and ceftiofur: 96%) (Figure 3). Notably, KCJK5143 strain was one of the isolates having a MIC of cefotaxime higher than 256 μg/mL, and KCJK5106 isolate was resistant to all antibiotics except colistin. Both two strains were isolated from the farm 18, where the farm indicated the highest prevalence of CTX-M/CMY-2 producing *E. coli* (Figure 1C).

### Identification of Antimicrobial Resistance Genes and Virulence Profiles

**FIGURE 4:**
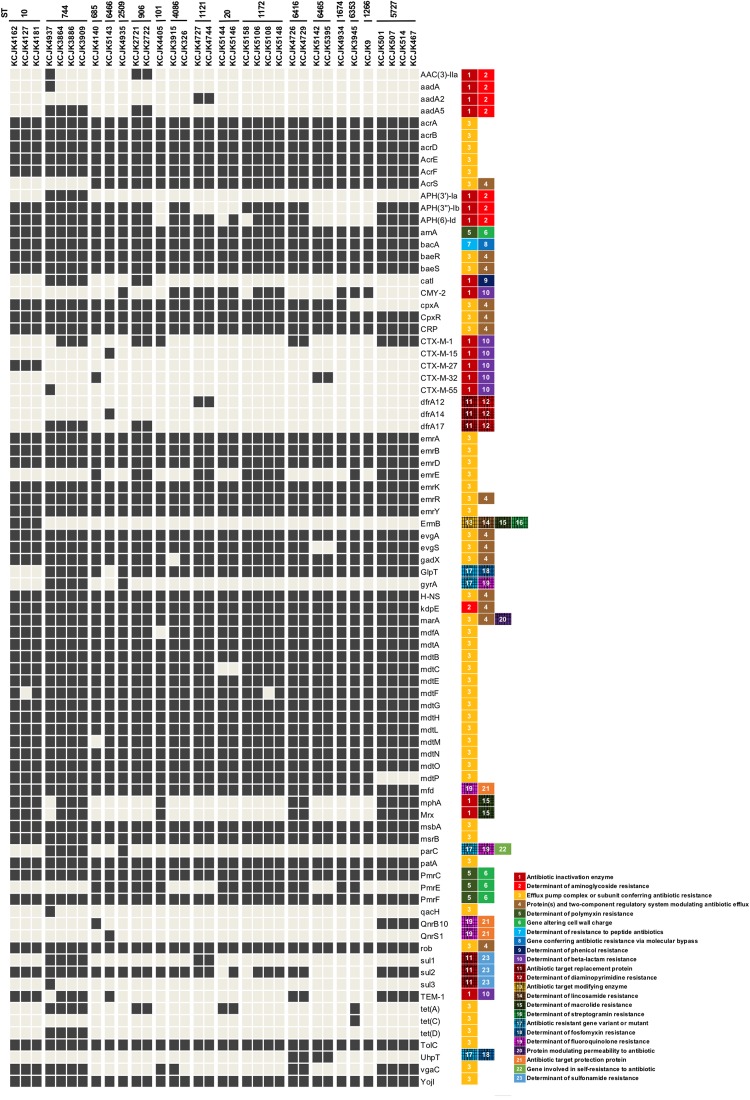
Distribution of antimicrobial resistance genes in representative isolates Thirty-four representative isolates were selected based on farm location, sample type, and sequence homology and identified ARGs using CARD database. Only ARGs that indicated the identity higher than 95% were included. The classification of ARGs was based on the CARD database and listed next to ARGs. Black and gray squares indicate the presence and absence of ARGs, respectively.

Out of 59 isolates, we selected 34 representative isolates for further studies based on the similarity of genome structure, originated farms and sample types (Figure 2 and Supplementary Figure S1). The profile of ARGs indicated that the representative isolates harbored multiple ARGs, which consisted 12 classes of ARGs (i.e., aminoglycoside, polymyxin, peptide antibiotics, phenicol, β-lactam, diaminopyrimidine, lincosamide, macrolide, streptogramin, fosfomycin, fluoroquinolone, and sulfonamide) and efflux pump complex. Isolates with same ST indicated similar ARG profiles, and all of the isolates contained polymyxin (*arnA*, *pmrC*, and *pmrF*), peptide antibiotics (*bacA*), fluoroquinolone (*mfd*) resistance genes, and efflux pump systems as well as β-lactamase genes (CMY-2, CTX-M, or TEM), regardless of their ST (Figure 4), coinciding with the AST result, which showed that all isolates were multidrug resistant.

**FIGURE 5:**
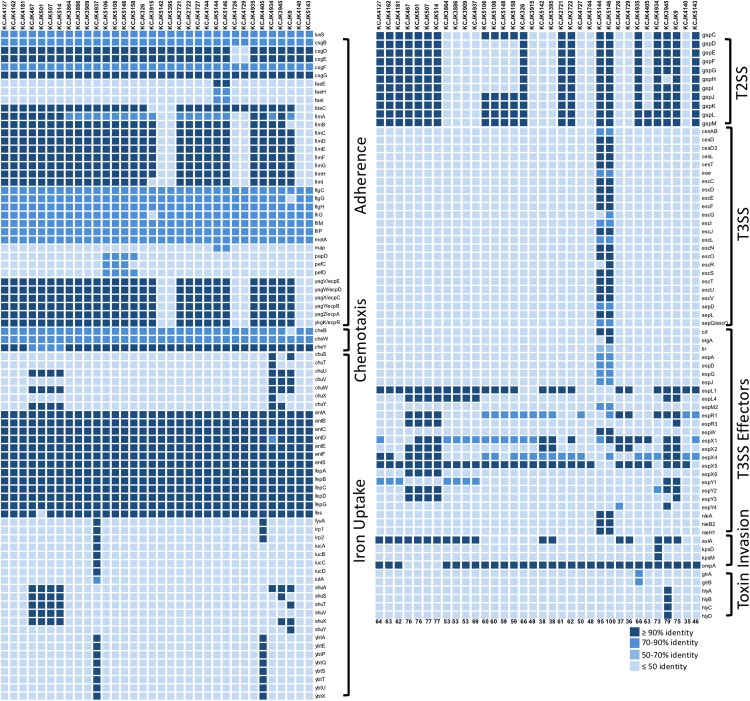
Profile of virulence factors in representative isolates Virulence factors (VFs) in the 34 representative isolates, selected by the similarity of genome structure, source of farms, and sample types, were identified using VFDB. Protein sequences of the representative isolates (query sequences) were aligned to the reference sequences (subject sequences) in VFDB using BLASTp, and only the proteins that indicated query and subject coverage higher than 50% were included in the figure. Depending on the function of each VF, VFs were classified, and gradation color indicates the identity of VFs compared to the reference sequences. The listed numbers at the bottom indicate the number of identified VFs in each isolate.

In addition to the ability of antibiotic resistance, the isolates had versatile virulence factors (VFs). VFs associated with adherence, chemotaxis, iron uptake, type II (T2SS) and III (T3SS) secretion systems, invasion, and toxin were identified (Figure 5). Notably, adherence (flagella; *flgCGH*, *fliGMP* and curli; *csgBFG*), chemotaxis (*cheBWY*), and iron uptake (enterobactin; *entABCDEFS*, *fepABCDG*) VFs were common across all the representative isolates (Figure 5). The number of VFs ranged from 35 to 100. Mostly, phylogroup B2 and D had higher number of VFs compared to A and B1 groups with a few exceptions as previously reported (Salipante et al., 2015). Some isolates (KCJK5144, KCJK5146, and KCJK3945) were belonging to phylogroup B1, but it showed higher number of VFs unlike other phylogroup B1 strains. In particular, VFs associated with extraintestinal pathogenic *E. coli* (ExPEC) were found from our isolates (Pitout, 2012), including *fimH* (type I fimbriae: adhesins), *kpsM* (capsule), *iutA* (siderophore receptor), *fyuA* (siderophore receptor), and *hlyD* (hemolysin: toxin), even though the isolate with all above VFs at the same time was not identified. KCJK5144 and KCJK5146 encoded T3SS and effector proteins containing Tir (translocated intimin receptor), Nle (non−LEE encoded effectors), EspG (*Escherichia* secretion protein), and Cif (cycle inhibiting factor). KCJK5144 and KCJK5146 strains contained 95 and 100 VFs respectively, which were the highest number of VFs among the 34 representative isolates (Figure 5).

### Adhesion of CTX-M and CMY-2 Producing *E. coli* to Human Cells

**FIGURE 6:**
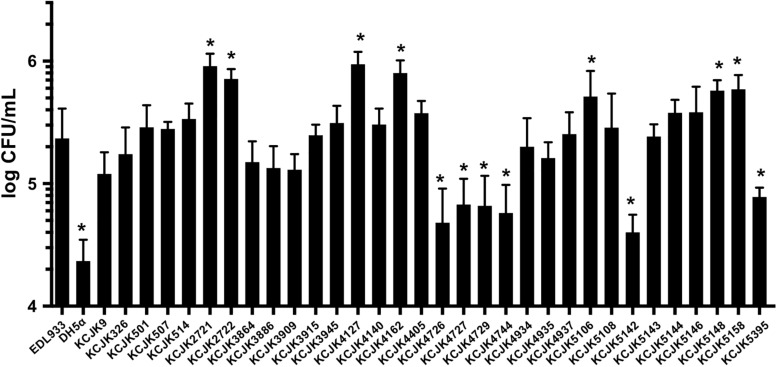
Adhesion of representative isolates to Caco-2 cells Thirty-two representative animal isolates were inoculated to Caco-2 cells, and EDL933 and DH5α were used as positive and negative controls, respectively. Bar graphs indicate the mean of four measurements, and the error bars represent standard deviation of the measurements. An asterisk means significant differences compared to EDL933.

We found all of the ESBL and AmpC producing *E. coli* isolates shared VFs associated with adherence through *in silico* analysis (Figure 5). To evaluate adhesion property of CTX-M and CMY-2 positive *E. coli* isolates with *in vitro* biochemical assays, adherence assay was conducted with human colon carcinoma cell line (Caco-2). The adherence ability of the representative strains from animal sources was compared with EDL933 as a positive control and DH5α as a negative control (Figure 6). Out of 32 isolates, seven and six isolates showed significantly high and low adhesion respectively, compared to EDL933 (*P* < 0.05) (Figure 6). Most of the isolates (59%, 19/32) were comparable with EDL933, but significantly higher than DH5α (*P* < 0.05). Notably, KCJK5106, KCJK5148, and KCJK5158, which showed higher adherence ability, were harboring more adherence VFs such as *papD*, *pefC*, and *pefD* (Figures 5, 6). KCJK4726, KCJK4729, KCJK5142, and KCJK5395 strains, which showed lower adherence, did not contain fimbriae genes (*fimABCDEFGHI*) and *E. coli* common pilus (ECP) genes (*ecpABCDE*), both of them are necessary for the effective adhesion (Figures 5, 6) (Alcántar-Curiel et al., 2013; Li et al., 2018).

### Functional Genomic Analysis

**FIGURE 7:**
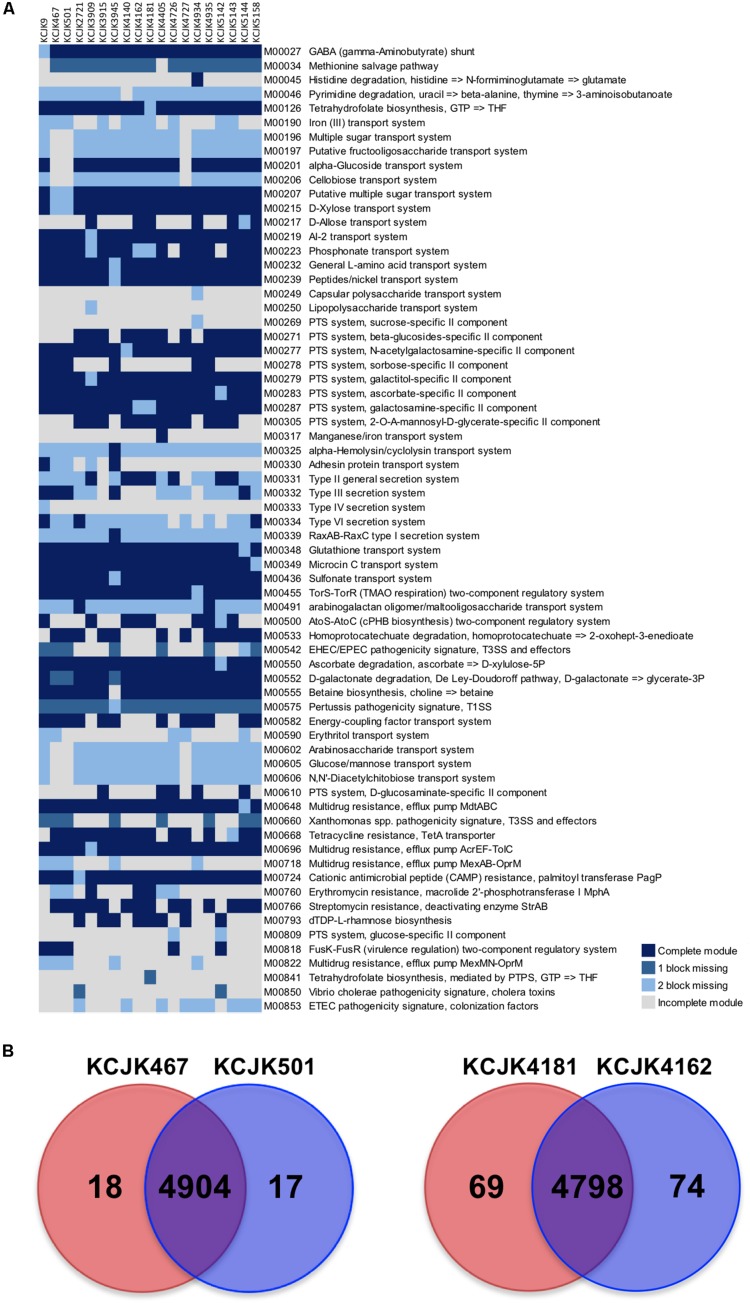
Comparison of KEGG modules and CDS **(A)** KEGG modules of the 19 representative isolates from cattle feces, forage, and soil were identified and compared. The identified KEGG modules were classified into four categories, including complete module (block in dark blue), module with one block missing (block in blue), module with two blocks missing (block in light blue), and incomplete module (block in light gray). Only the modules showing different categories among all isolates were shown in a heat map, with the module IDs, and functions listed to the right of the blocks. **(B)** The coding sequences (CDS) of fecal and environmental isolates were compared to investigate the similarity of proteins. The CDS numbers of environmental and fecal isolates were listed in red and blue circles, respectively. The number in a red or blue shadow present the number of unique CDS in each isolate, while the number in the overlapped shadow indicates the orthologous proteins shared by two isolates.

To investigate potential differences of functional capability between fecal and environmental isolates, we identified and compared the KEGG functional modules of the 19 isolates from 17 different STs, including the isolates from the cattle feces, forage, and soil. The KEGG functional modules were classified into four catagories, including complete module, module with one block missing, module with two blocks missing, and incomplete module. A total of 346 functional modules were identified. Among these modules, 278 modules were conserved in all isolates, while the other 68 modules were different among the isolates (Figure 7A). KCJK467, the forage isolate, and KCJK501, the fecal isolate, harbored almost identical functional modules, except for the module related to erythritol transport system (M00590). Similarly, KCJK4181, the soil isolate, and KCJK4162, the fecal isolate, carried highly similar functional modules with only the difference in tetrahydrofolate biosynthesis (M00126 and M00841).

To further investigate the similarity of these two pairs of isolates, we compared individual proteins in each isolates by identifying orthologous proteins. A total of 4,904 CDS were identified as orthologous proteins shared by KCJK467 and KCJK501, while 18 and 17 CDS were unique in KCJK467 and KCJK501, respectively (Figure 7B). These unique CDS were mostly related to hypothetical proteins (Supplementary Table S1). KCJK4162 and KCJK4181 had 4,798 common CDS, as well as 74 and 69 unique CDS, respectively (Figure 7B). The functions of the unique CDS were hypothetical proteins and *IS1* family transposase (Supplementary Table S2). The comparison result of CDS between fecal and environmental isolates are concordant to the comparison result of the KEGG functional modules that fecal and environmental isolates are quite similar, underlining the capability of ESBL-producing *E. coli* to be transmitted between different niches.

### Characterization of CTX-M and CMY-2 Genes on *E. coli*

All sequenced *E. coli* isolates had either CMY-2, CTX-M, or TEM types of β-lactamase genes (Table 3). The most prevalent gene type was CTX-M (39/59; 66.10%), and the CTX-M types included CTX-M-1 (*n* = 22), CTX-M-15 (*n* = 1), CTX-M-27 (*n* = 12), CTX-M-32 (*n* = 3), and CTX-M-55 (*n* = 1). CMY-2 was present in 33.89% of the isolates (20/59), and 54.23% (32/59) isolates encoded TEM-1A or TEM-1B genes. Especially, the isolates with TEM gene co-harbored CTX-M gene (Table 3). Most of them encoded β-lactamase genes in their plasmid, and only four isolates (6.7%) included the genes in their chromosomal DNA. All of the four isolates had same ESBL gene type as CTX-M-32 (Table 3). The most prevalent plasmid type in all isolates was IncF and IncR groups, and other Inc groups, including IncX, IncP, IncA/C2, IncY, IncHI, IncQ, IncI, and IncB/O/K/Z, were also found. As another plasmid type, there were Col156, ColRNAI, ColMG828, and ColpVC (Table 3).

**FIGURE 8:**
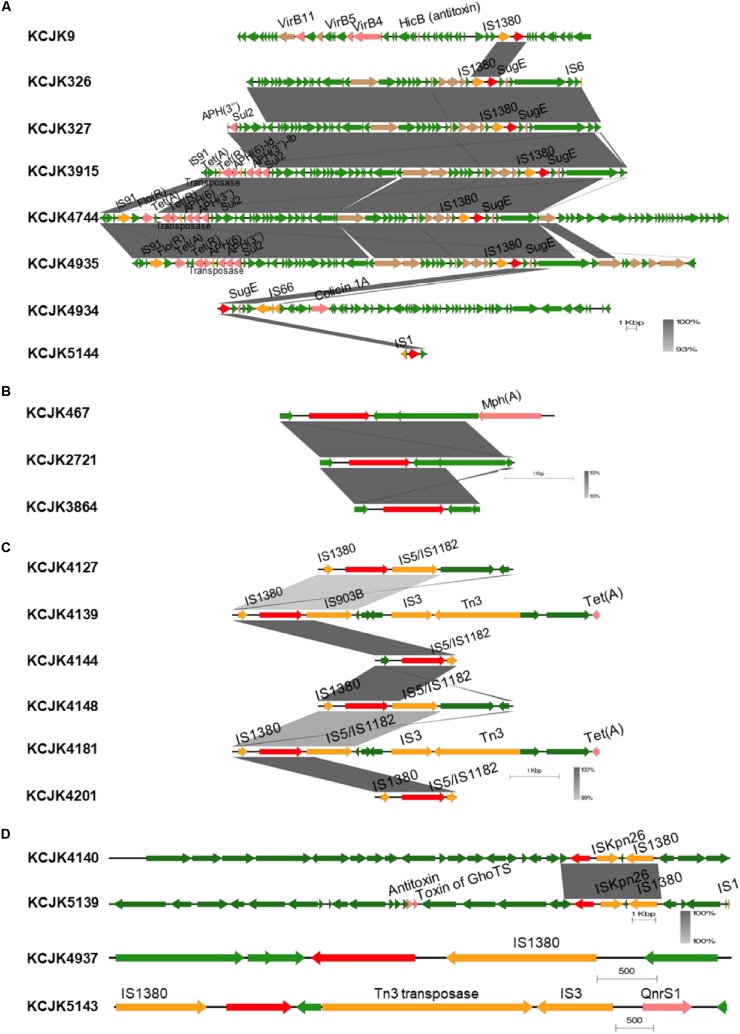
Genetic environment of β-lactamase genes in representative isolates Linear map shows the regions surrounding β-lactamase genes in each isolate. Isolates encode CMY-2 **(A)**, CTX-M-1 **(B)**, CTX-M-27 **(C)**, and CTX-M-32, CTX-M-15, and CTX-M-55 **(D)** are presented. Only isolates showing different structures were included, and sequence homology was compared if the isolates carried same β-lactamase gene. Gradient-color between linear maps indicates the similarity between isolates. β-lactamase genes, other virulence factors, insertion sequences, conjugal transfer genes, and general CDS are indicated by red, pink, yellow, brown, and green arrows, respectively.

Interestingly, genetic environment of β-lactamase genes showed high similarity between isolates, even though the isolates were isolated from different farms (Figure 8 and Supplementary Figures S2A–E). Depending on their β-lactamase gene types, we divided the isolates into different groups (Figures 8A–D) and compared the contigs harboring β-lactamase genes (Supplementary Figures S2A–E). Regardless of their sources, genetic environment of β-lactamase genes showed the high homology if the isolates had same β-lactamase gene type (Figure 8 and Supplementary Figures S2A–E). Although the isolates in CTX-M-1 group were not identified any IS due to the short length of contigs (Figure 8B), most isolates in other groups, such as CMY-2, CTX-M-27, CTX-M-32, CTX-M-15, and CTX-M-55, carried *IS1380* family insertion sequences in common, and the *IS1380* element was located upstream of CTX-M and CMY-2 genes (Figures 8A,C,D). In particular, the isolates encoding CMY-2 gene harbored IS in the upstream and *sugE* gene in the downstream as previously reported (Singh et al., 2018). Furthermore, the set of *tra* genes related to conjugal transfer system was co-identified in the isolates with CMY-2 (Figure 8A). Other virulence genes associated with ARGs and toxins were located near β-lactamase genes as well. These results suggest that wide spread of β-lactamase genes may be caused by the existence of IS and conjugation systems, and additional virulence genes could be acquired at the same time when β-lactamase genes are transferred to others through horizontal gene transfer (Yamashita et al., 2014).

### Global Transmission of Commercial Farm Isolates

**FIGURE 9:**
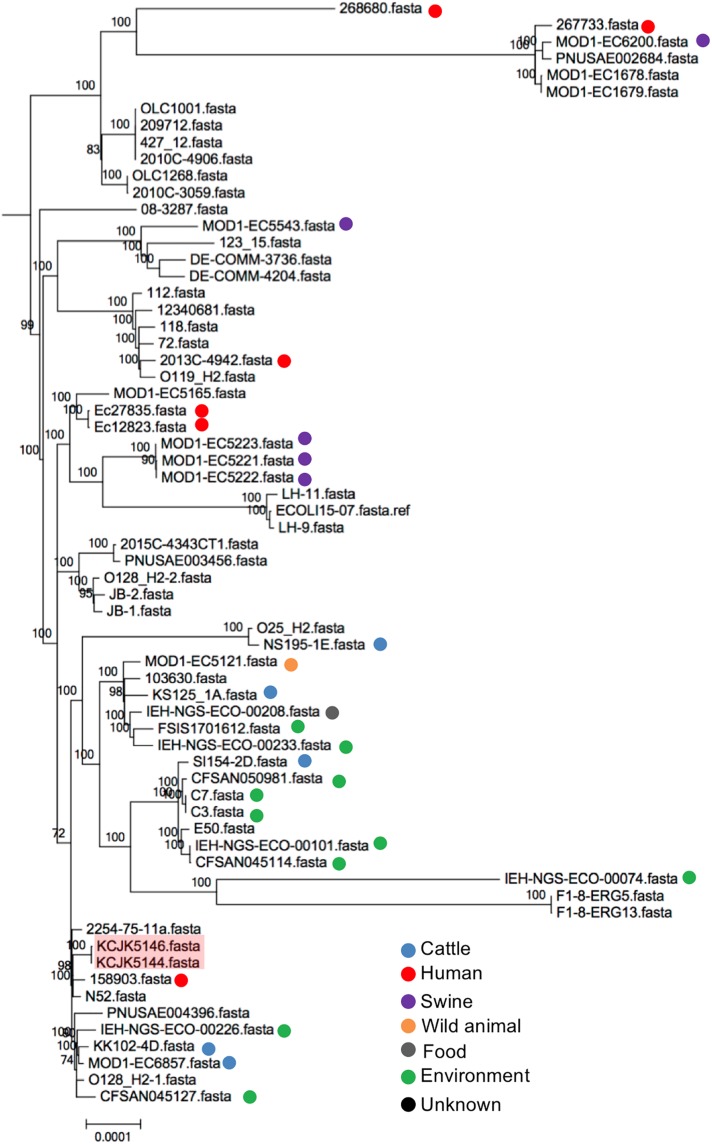
Phylogeny of ST20 *E. coli* isolates The phylogenetic tree with ST20 *E. coli* isolates was generated with Parsnp software based on core-genome SNPs. Reference sequences (*n* = 64) were available in Enterobase. Strains isolated from this study were shaded in red, and the reference strains were indicated by different colored dots based on the originated sources.

To compare genetic relationship between our isolates and global strains from diverse sources, a phylogenetic tree was analyzed to understand the originated sources. Depending on the MLST, we generated phylogenetic trees with relevant strains available on Enterobase, a database of *E. coli* with metadata and genotypes based on whole genome assemblies (Figure 9 and Supplementary Figures S3A–O) (Tagini and Greub, 2017). Out of 17 different ST, ST6465 included only our isolates, thus, we excluded ST6465 to generate a phylogenetic tree. Other 16 ST contained the isolates originated from diverse sources (i.e., human, cattle, swine, wild animals, food, and environment) and countries. The isolates used in this study were closely clustered with the reference strains, however, they were not clonal variants (Supplementary Figures S3A–O). Some isolates had relatively small number of SNPs, indicating that the isolates may have a same ancestor strain experiencing spontaneous genetic changes, gene transfer, or evolutionary processes in different areas (Guenther et al., 2012; Livermore, 2012; Watson et al., 2012). For instance, there were 2,283 SNPs between KCJK5144, isolated from cattle in this study, and 158903 that was originated from human in the United Kingdom (Figure 9). Furthermore, KCJK5144 strain showed close genetic distances with cattle and environmental strains from other countries as well, suggesting the isolate could be transmitted from outside sources through migrated birds or direct human contacts.

## Discussion

All commercial cow/calf operations tested in this study contained either CTX-M or CMY-2 positive bacteria, and more than half of the farms carried both CTX-M and CMY-2 producing *E. coli*. The prevalence and concentration of CTX-M or CMY-2 genes were high in environmental samples, and environmental isolates were closely related to isolates from cattle feces. By comparing genome sequences, it was more likely that these bacteria were transmitted between farms in North Central Florida, as well as arising independently as clones within farms. In general, antibiotic use in animals is known to accelerate the advent of antibiotic resistant bacteria (Smith et al., 2002). In addition to antibiotic usage, the selection pressure during the process of evolution in microbial communities could be another reason for naturally occurring antibiotic resistant bacteria. Recent studies suggested that genomic breed composition of cattle was also associated with different gut microbiota structure (Fan et al., 2019a) that affects the colonization of antibiotic resistant bacteria (Fan et al., 2019b). In these cases, the prevalence of antibiotic resistant bacteria might not be directly correlated with antibiotic usage, and even farms without antibiotic supplementation contained ESBL and AmpC producing bacteria in both environmental and fecal samples (Mir et al., 2016, 2018; Teng et al., 2019), coinciding with the results of our study (Figures 1B,D). Remarkably, some farms evaluated in this study did not have CTX-M or CMY-2 producing *E. coli* (i.e., farm 5, 6, 7, 13, 15, and 16), even though these farms were geographically adjacent to the farms carried CTX-M or CMY-2 producing *E. coli* (Figure 1). Given the antibiotic resistance mitigation strategies suggested by Ma et al. (2019), it is plausible that farm managements such as animal herd size, feeding practices, and farm hygiene might have resulted the differences in the prevalence of CTX-M/CMY-2 producing *E. coli* in these farms. Further investigation seeking the factors that reduce the prevalence of CTX-M/CMY-2 producing *E. coli* will be needed to develop effective mitigating strategies for antimicrobial resistance.

Most ESBL and AmpC β-lactamase genes are carried through plasmid DNA, thus, the genes can be easily acquired, deleted, and evolved between bacteria through horizontal gene transfer. Except for CTX-M-32, other ESBL genes were encoded in plasmid, and IncF and IncR were the major identified plasmids across the isolates. IncF has been reported that it carries various resistance genes of aminoglycoside, quinolone, and efflux pumps (Carattoli, 2009), and IncR plasmid also harbors resistant genes to many antibiotics including β-lactams, sulfonamides, quinolones, aminoglycosides, tetracyclines, chloramphenicol, and trimethoprim (Rozwandowicz et al., 2018). Our results also showed resistance ability of CTX-M and CMY-2 producing *E. coli* against clinically important antibiotics, which consisted of the class of aminoglycoside, penicillin, polypeptide, quinolone, sulfonamide, tetracycline, and cephalosporin. This suggests that the *E. coli* encoding CTX-M and CMY-2 genes isolated from food animals could pose a risk to humans if zoonotic transmission occurs (Nikaido and Pagès, 2012; Wellington et al., 2013).

Among many types of CTX-M genes, CTX-M-15-producing ST131 *E. coli* is known as one of the most widely disseminated strains, and these strains have become a major problem causing many human cases of infections (Petty et al., 2014). These isolates are multidrug resistant and have virulence determinants with a high potential virulence compared to other CTX-M types of *E. coli* (Coque et al., 2008b; Coelho et al., 2011). The strain KCJK5143 was identified as CTX-M-15-producing *E. coli* and was assigned to ST6466. The strain had MIC of 256 μg/mL against cefotaxime which is far greater than the clinically permitted concentration of cefotaxime (64 μg/mL). In addition to *E. coli* with CTX-M-15, more than half of the isolates had higher MIC of cefotaxime than 64 μg/mL.

Most of the studies related to MLST of ESBL-producing *E. coli* have focused on the specific sequencing type (i.e., ST131), limited ESBL genes such as CTX-M-15, or focuses on clinical isolates, whereas the genomic characteristics of animal strains are studied less often. Non-biased studies are required to demonstrate the relatedness between human and animal isolates and to understand the genetic characteristics of ESBL-producing *E. coli* isolated from diverse hosts (Oteo et al., 2009). CTX-M-1 was the major type of CTX-M gene in this study. However, except for CTX-M-1, the distribution of other CTX-M types was not overlapped with Europe and Asia, showing that the distribution of CTX-M genes in *E. coli* is dependent on geographical areas (Brigante et al., 2005). ST10 was the predominant MLST, and ST10 has been reported from clinical samples indicating the ST10 isolates can colonize in humans as well as animal (Wang et al., 2016). Recent study also found that ESBL-producing *E. coli* ST10 occurred in both humans and cattle, and CTX-M-14 was the predominant CTX-M type among the bovine isolates (Day et al., 2019). In the central area of Spain, ESBL-producing *E. coli* ST10 was disseminated carrying CTX-M-14 (Coque et al., 2008a), and another study showed that ST10 *E. coli* carried CTX-M-14, SHV-12, CTX-M-9, CTX-M-15, and CTX-M-32 (Oteo et al., 2009). In our study, strains with ST10 harbored CTX-M-27 and TEM-1A, showing that sequencing types are associated with many ESBL genes not only a specific gene.

All isolates contained various VFs linked with adherence, chemotaxis, iron uptake, and bacterial secretion systems, and most of them have adhesion ability to human cells comparable to EDL933 that uses high adherence ability to cause diseases in host (Figures 5, 6). In particular, some isolates (KCJK5144 and KCJK5146) carried a pathogenicity island, the locus of enterocyte effacement (LEE) harboring the gene clusters of T3SS, adhesion, intimin, the translocated intimin receptor, and secreted effector proteins, showing the characteristics of enteropathogenic *E. coli* (EPEC) (Wong et al., 2011). Serotype of KCJK5146 was O128ac:H2 which is known as one of the most frequent serotypes of atypical EPEC (Alonso et al., 2016). Furthermore, KCJK5144 and KCJK5146 strains were clustered with human isolate having a small number of SNPs (Figure 9), suggesting that these ESBL/AmpC producing *E. coli* isolated from food animals could cause human diseases that would be resistant to multiple antibiotics. We can also identify VFs related to ExPEC strain from our isolates. Although most of ExPEC strains are limited to phylogroups B2 and D (Dale and Woodford, 2015), our isolates containing VFs related to ExPEC strains were not allocated as B2 or D only. Phylogroup A and B1 *E. coli* isolates also contained VFs associated with ExPEC, as previously reported (Valat et al., 2012).

ESBL/AmpC producing *E. coli* isolates were closely clustered with other global strains, showing the isolated strains in this study were not raised within specific area. Even though we cannot find clonal variants from reference sequences available on Enterobase, our isolates made mixed clusters with the reference strains, showing the genetic similarity between animal and clinical isolates (Platell et al., 2011). It is still challenging to identify vehicles and transmission routes despite the findings of transmission of ESBL/AmpC producing *E. coli* between farms (Wu et al., 2013). Controlled experiments to verify the transmission are necessary to understand the possible transmission routes mitigating rapid dissemination of ESBL/AmpC producing *E. coli*. In addition, plasmid transmission among different reservoirs has been reported with high level of similarity and small number of SNPs between plasmids from animal and clinical isolates (de Been et al., 2014). In our study, similar genetic environment of CTX-M and CMY-2 producing *E. coli* isolates regardless of the originated farms also supported that horizontal gene transfer between bacteria had occurred, and plasmid transmission is likely to happen causing the widespread of ESBL genes. From plasmid typing results, we can find that plasmid types were overlapped within farm area (Table 3). For further studies, plasmid sequences of ESBL/AmpC producing *E. coli* isolates could be analyzed to verify the relationship of plasmids among different isolates and the distribution of other ARGs located in plasmids.

In the current study, we investigated the presence of multi-drug resistant bacteria among cattle and the environment using whole genome sequencing and traditional techniques to identify both genotypic and phenotypic characteristics. Our research provides a detailed understanding of CTX-M and CMY-2 producing *E. coli* isolated from beef cow/calf production systems where animals are raised without antibiotic supplementation. This study demonstrates that even farm animals with seldom use of antibiotics serve as reservoirs of ESBL/AmpC producing bacteria and provide a possible zoonotic transmission into human populations. Our research highlights the need for further investigation of ESBL/AmpC producing *E. coli* in beef cattle and the implementation of diverse monitoring and control strategies in an effort to mitigate the global transmission of such genes to humans and health care systems.

## Data Availability Statement

The datasets generated for this study can be found in the NCBI database under the BioProject PRJNA298331.

## Ethics Statement

The animal study was reviewed and approved by the University of Florida, Institutional Animal Care and Use Committee (IACUC protocol #201308027).

## Author Contributions

SL, LT, ND, TW, and KJ designed the study and drafted the manuscript. SL, LT, ND, and KJ collected the samples. SL and LT analyzed the data. SL and KJ finalized this manuscript.

## Conflict of Interest

The authors declare that the research was conducted in the absence of any commercial or financial relationships that could be construed as a potential conflict of interest.

## Footnotes

1 http://tree.bio.ed.ac.uk/software/figtree

2 https://enterobase.warwick.ac.uk

## Supplementary Material

FIGURE S1 Sequence similarity between isolates with the same ST. Based on the ST, whole genomes of the isolates were compared using the Mauve Aligner. The representative isolates for further studies were indicated with an asterisk (^∗^).

FIGURE S2 Genetic environment of CTX-M/CMY-2 genes in all isolates. Based on β-lactamase gene types, genetic environments of the isolates with (A) CMY-2, (B) CTX-M-1, (C) CTX-M-27, (D) CTX-M-32, (E) CTX-M-15 and CTX-M-55 were compared.

FIGURE S3 Phylogenetic tree of CTX-M/CMY-2 positive *E. coli* isolates with other global isolates. Phylogenetic analyses were conducted for the isolates with the same ST using Parsnp, including (A) ST10, (B) ST101, (C) ST685, (D) ST744, (E) ST906, (F) ST1121, (G) ST1172, (H) ST1266, (I) ST1674, (J) ST2509, (K) ST4086, (L) ST5727, (M) ST6353, (N) ST6416, and (O) ST6466. The isolates in each phylogenetic tree include the ones used in the study (with red background) and the global isolates obtained from Enterobase. The scale bars indicate the mean numbers of nucleotide substitution per site.

TABLE S1 List of unique CDS in KCJK467 and KCJK501 strains.

TABLE S2 List of unique CDS in KCJK4162 and KCJK4181 strains.
