# Supplementary material for: Prevalence and Molecular Characteristics of Extended-Spectrum and AmpC β-Lactamase Producing Escherichia coli in Grazing Beef Cattle
Source: Front Microbiol. 2020 Jan 9;10:3076. doi: 10.3389/fmicb.2019.03076 (PMC6962307; doi:10.3389/fmicb.2019.03076)
Supplement: FIGURE S1 — Sequence similarity between isolates with the same ST. Based on the ST, whole genomes of the isolates were compared using the Mauve Aligner. The representative isolates for further studies were indicated with an asterisk (∗). [file Presentation_1.PPTX]

## Slide 1
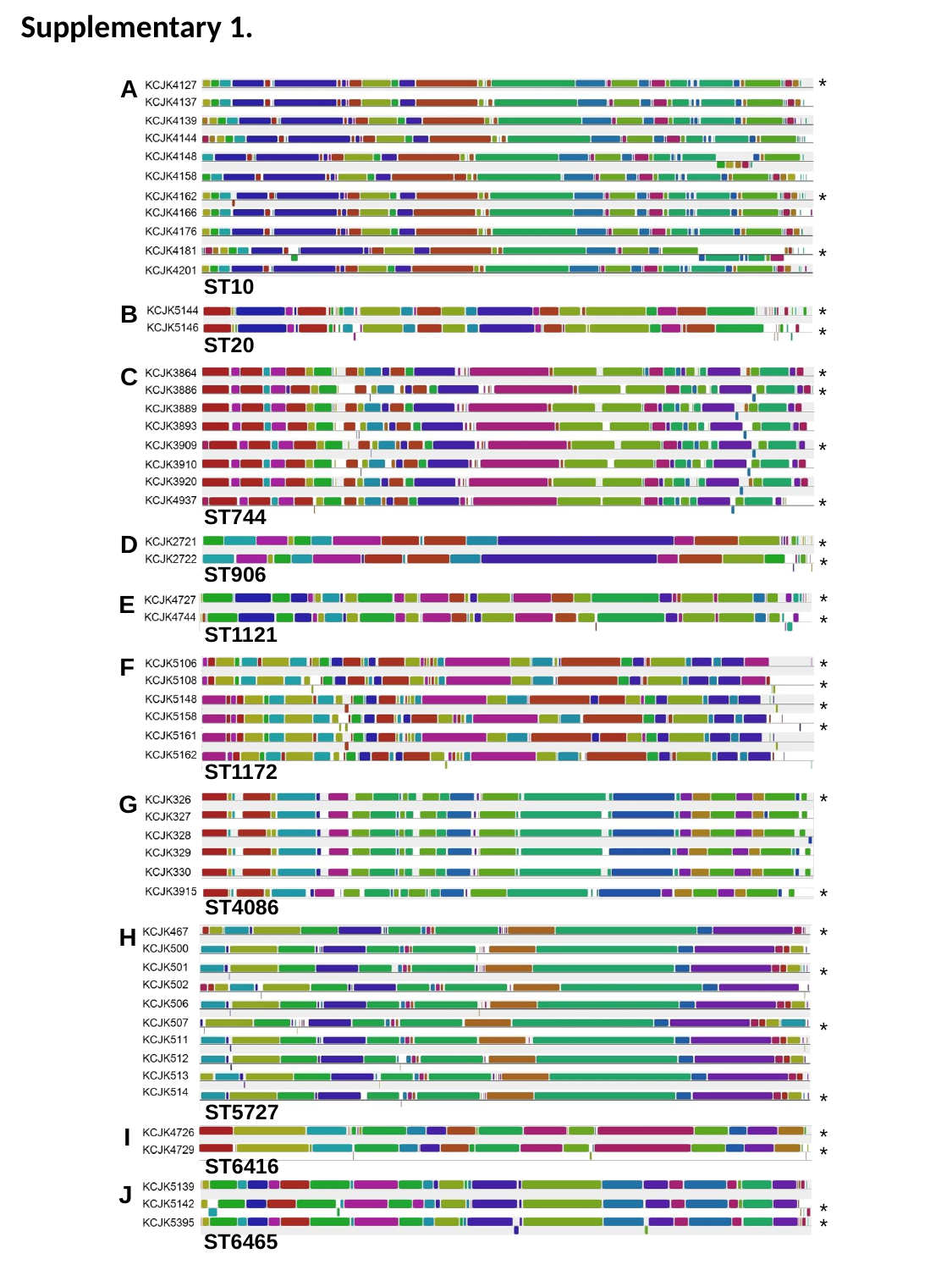

Supplementary 1.
*
A
*
*
ST10
B
*
*
ST20
C
*
*
*
*
ST744
D
*
*
ST906
*
E
*
ST1121
F
*
*
*
*
ST1172
*
G
*
ST4086
H
*
*
*
*
ST5727
I
*
*
ST6416
J
*
*
ST6465

## Slide 2
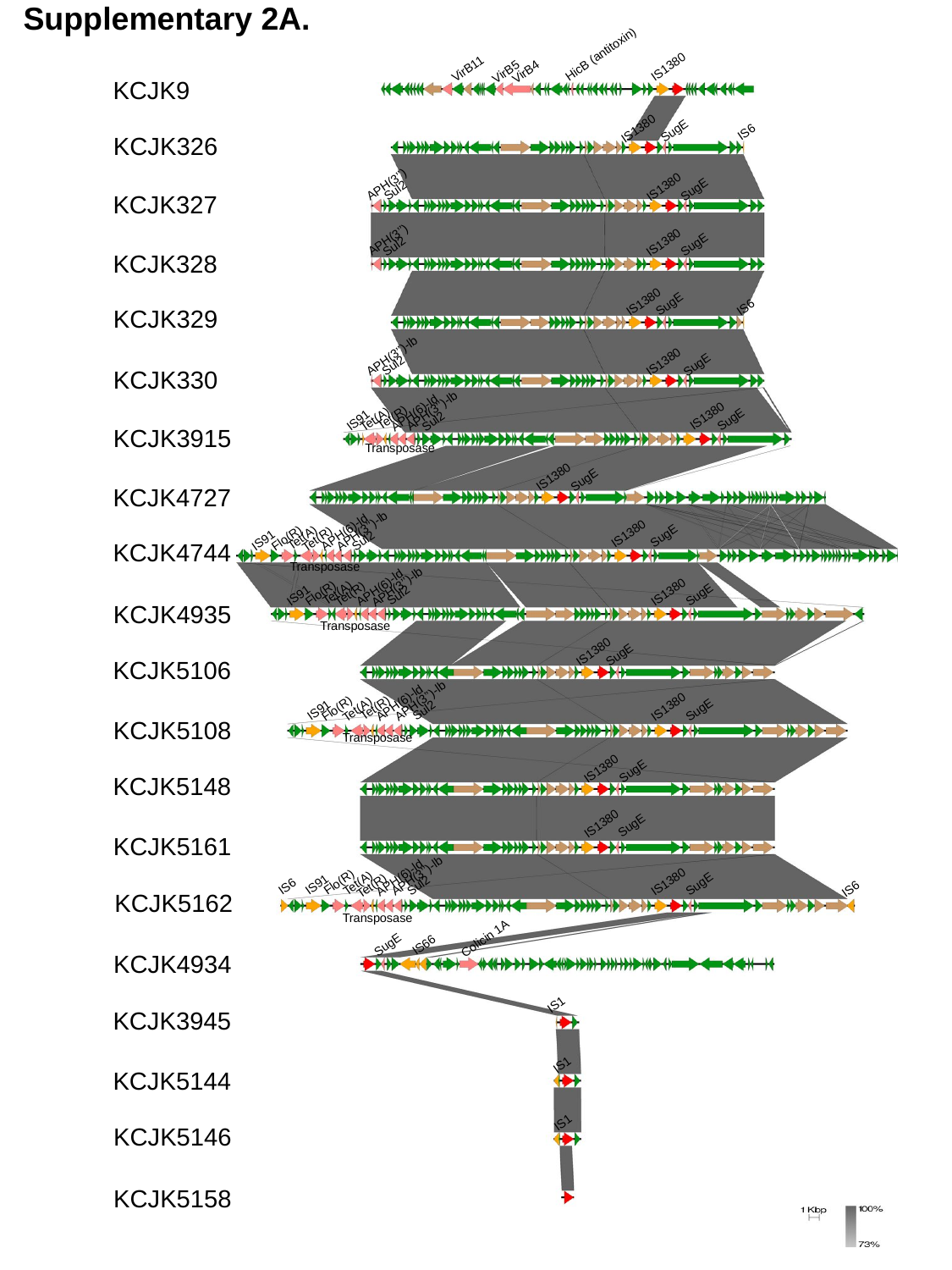

Supplementary 2A.
HicB (antitoxin)
IS1380
VirB11
VirB5
VirB4
KCJK9
IS1380
SugE
IS6
KCJK326
APH(3’’)
IS1380
SugE
Sul2
KCJK327
APH(3’’)
IS1380
SugE
Sul2
KCJK328
IS1380
SugE
IS6
KCJK329
APH(3’’)-lb
IS1380
SugE
Sul2
KCJK330
APH(3’’)-lb
APH(6)-ld
IS1380
Tet(R)
SugE
Tet(A)
IS91
Sul2
KCJK3915
Transposase
IS1380
SugE
KCJK4727
APH(3’’)-lb
APH(6)-ld
IS1380
SugE
Tet(A)
Flo(R)
Tet(R)
IS91
Sul2
KCJK4744
Transposase
APH(3’’)-lb
APH(6)-ld
IS1380
Tet(A)
Flo(R)
Tet(R)
SugE
Sul2
IS91
KCJK4935
Transposase
IS1380
SugE
KCJK5106
APH(3’’)-lb
APH(6)-ld
IS1380
Tet(R)
Flo(R)
Tet(A)
SugE
Sul2
IS91
KCJK5108
Transposase
IS1380
SugE
KCJK5148
IS1380
SugE
KCJK5161
APH(3’’)-lb
APH(6)-ld
IS1380
Flo(R)
Tet(A)
SugE
IS91
Sul2
Tet(R)
IS6
IS6
KCJK5162
Transposase
Colicin 1A
SugE
IS66
KCJK4934
IS1
KCJK3945
IS1
KCJK5144
IS1
KCJK5146
KCJK5158

## Slide 3
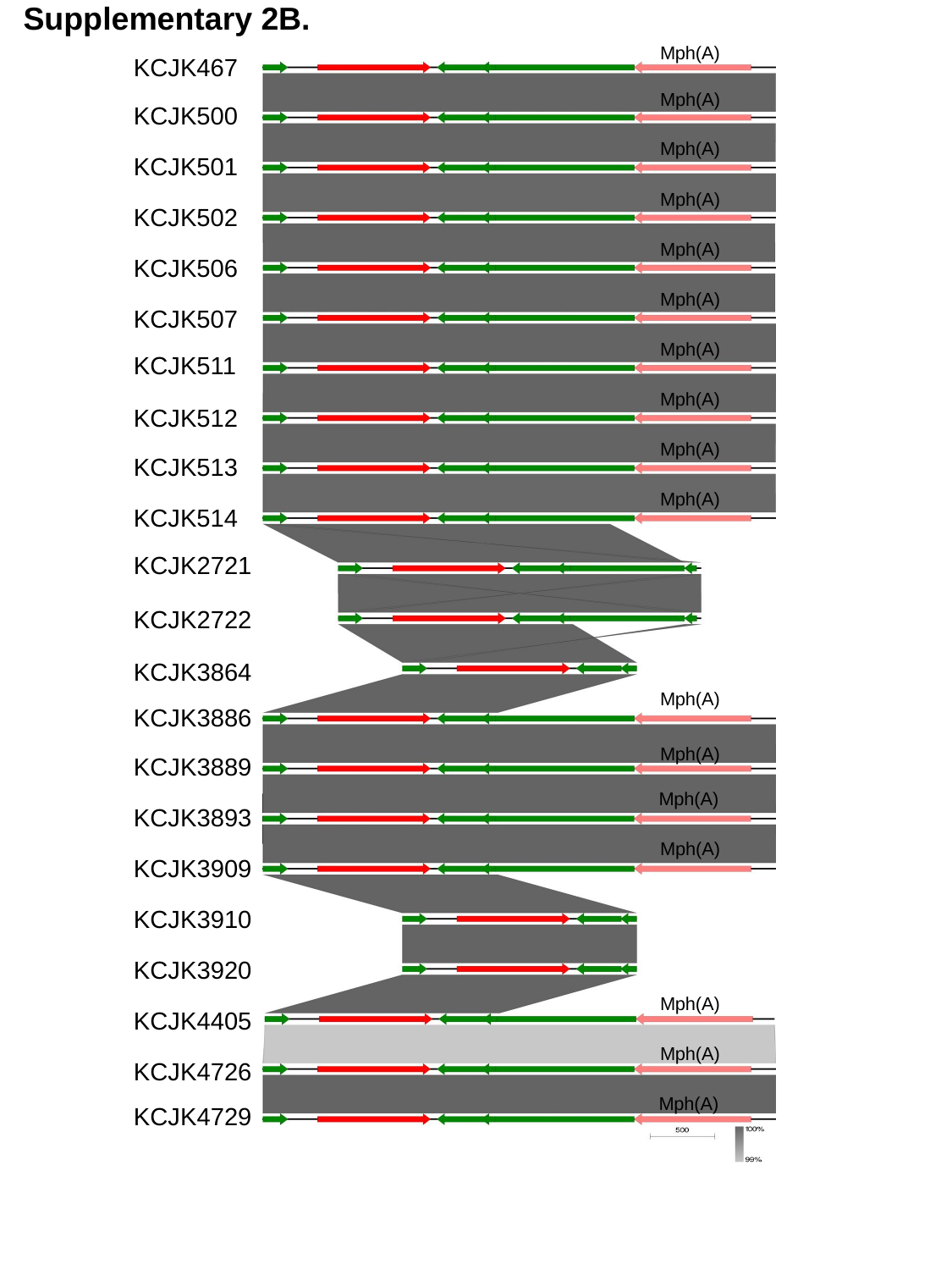

Supplementary 2B.
Mph(A)
KCJK467
Mph(A)
KCJK500
Mph(A)
KCJK501
Mph(A)
KCJK502
Mph(A)
KCJK506
Mph(A)
KCJK507
Mph(A)
KCJK511
Mph(A)
KCJK512
Mph(A)
KCJK513
Mph(A)
KCJK514
KCJK2721
KCJK2722
KCJK3864
Mph(A)
KCJK3886
Mph(A)
KCJK3889
Mph(A)
KCJK3893
Mph(A)
KCJK3909
KCJK3910
KCJK3920
Mph(A)
KCJK4405
Mph(A)
KCJK4726
Mph(A)
KCJK4729

## Slide 4
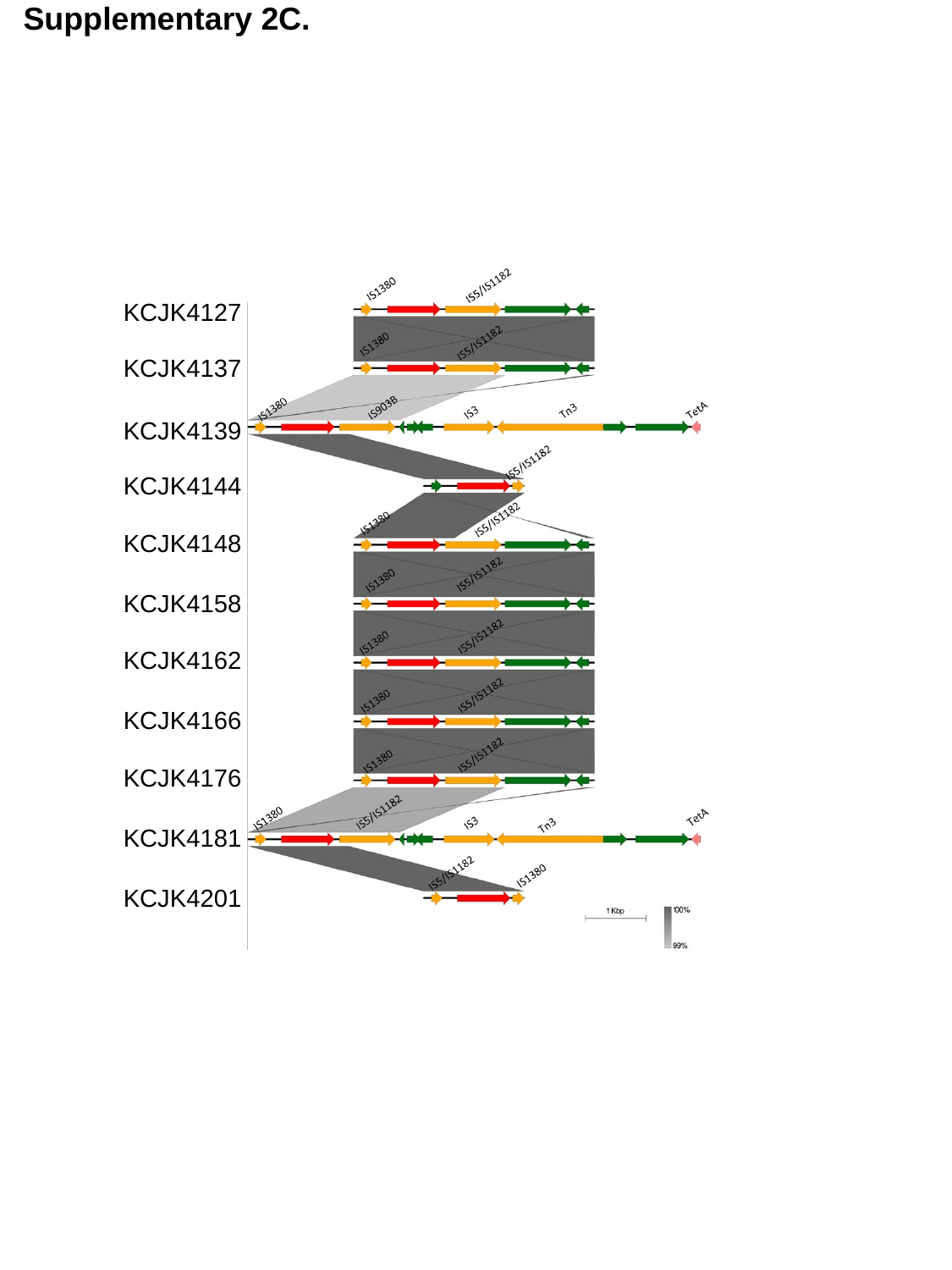

Supplementary 2C.
KCJK4127
KCJK4137
KCJK4139
KCJK4144
KCJK4148
KCJK4158
KCJK4162
KCJK4166
KCJK4176
KCJK4181
KCJK4201

## Slide 5
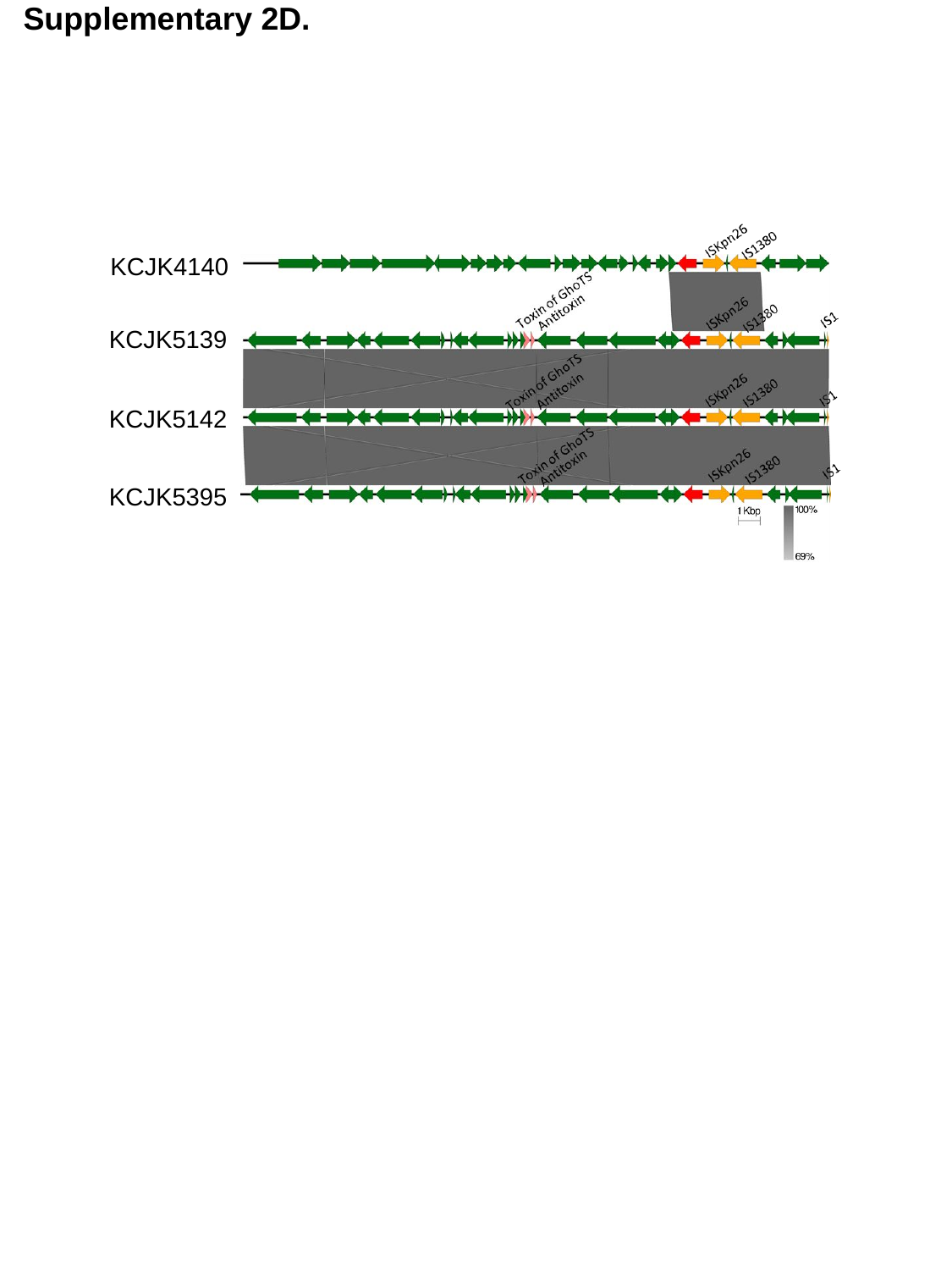

Supplementary 2D.
KCJK4140
KCJK5139
KCJK5142
KCJK5395

## Slide 6
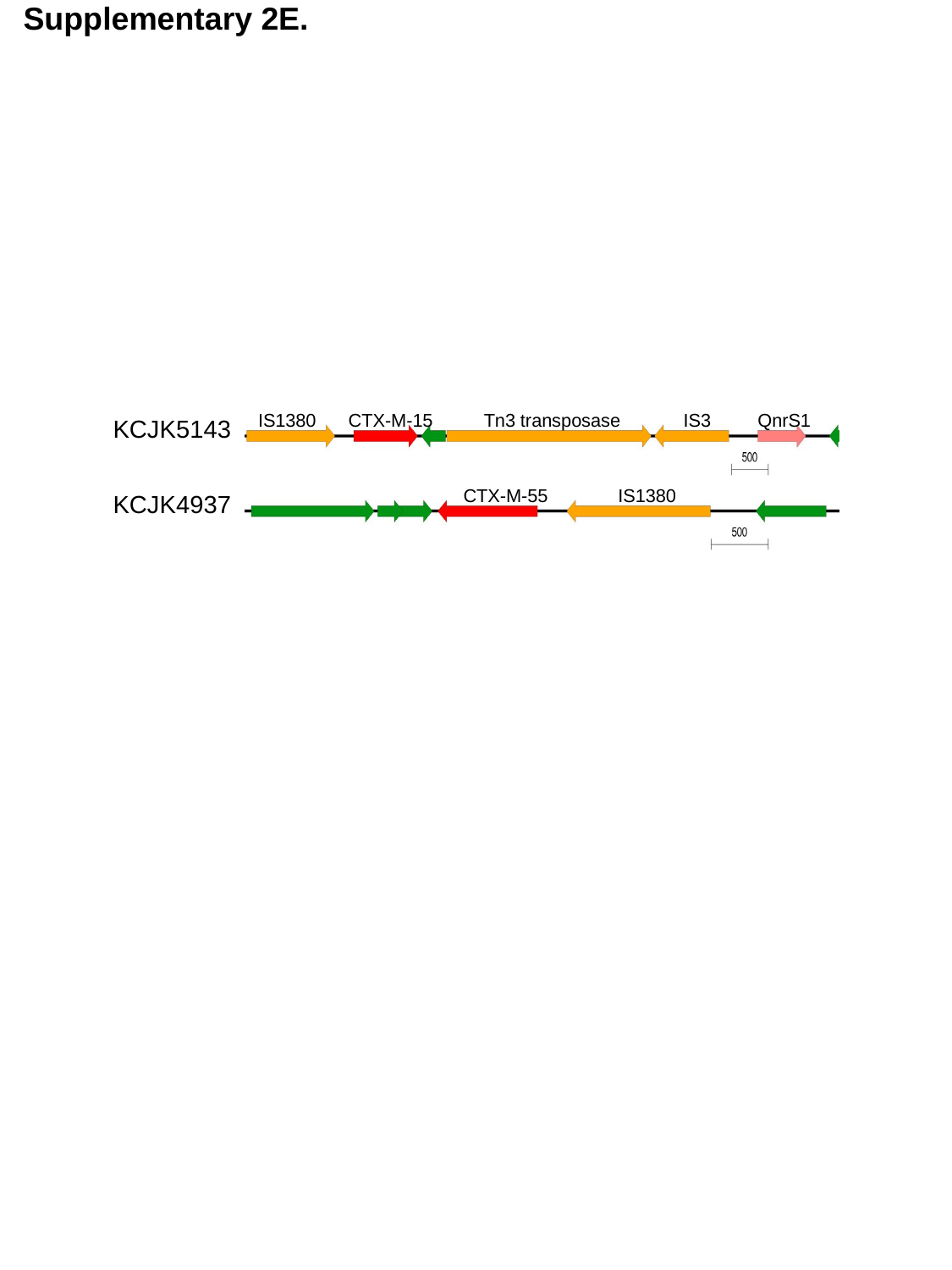

Supplementary 2E.
IS3
IS1380
CTX-M-15
Tn3 transposase
QnrS1
KCJK5143
CTX-M-55
IS1380
KCJK4937

## Slide 7
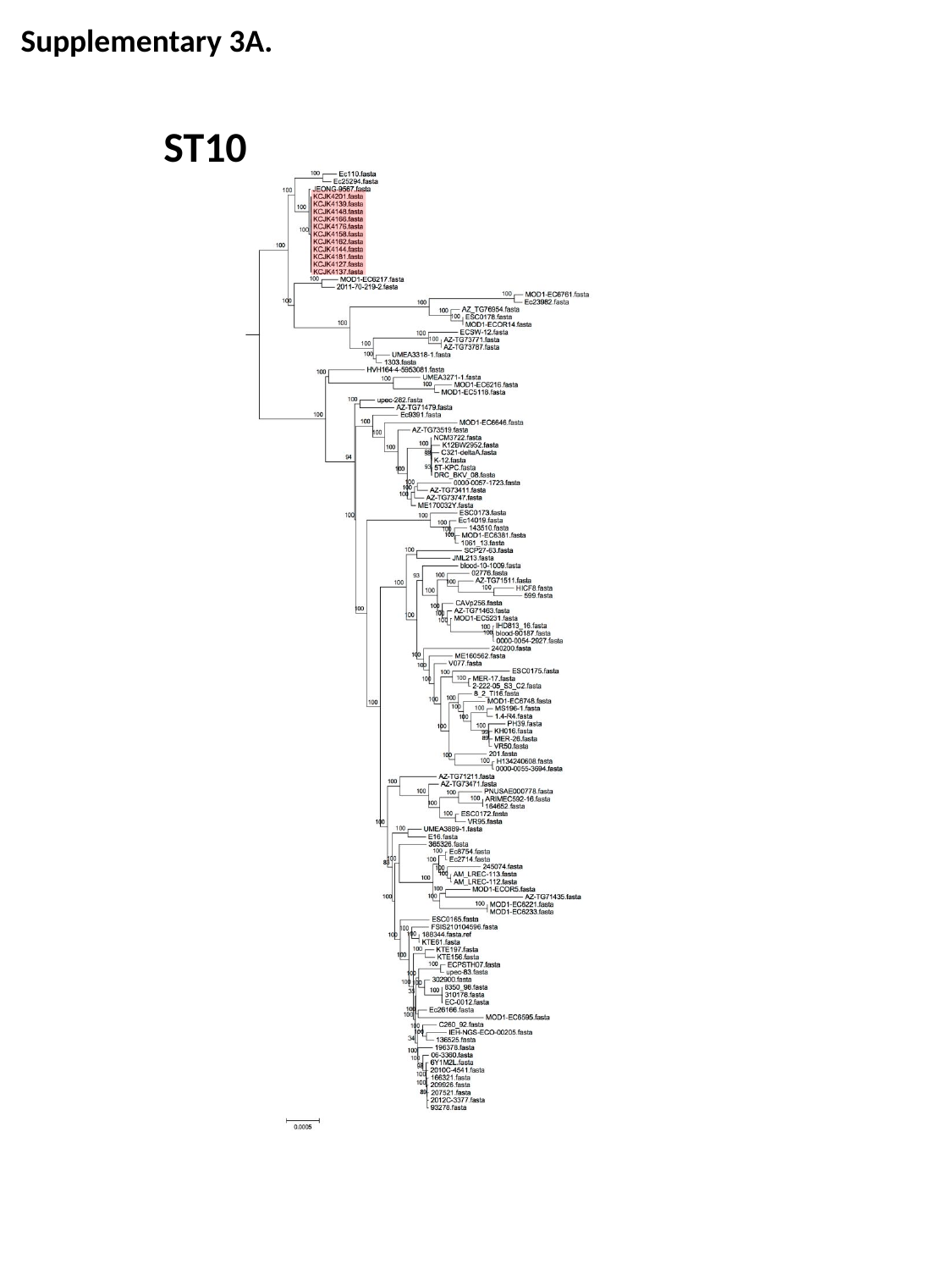

Supplementary 3A.
ST10

## Slide 8
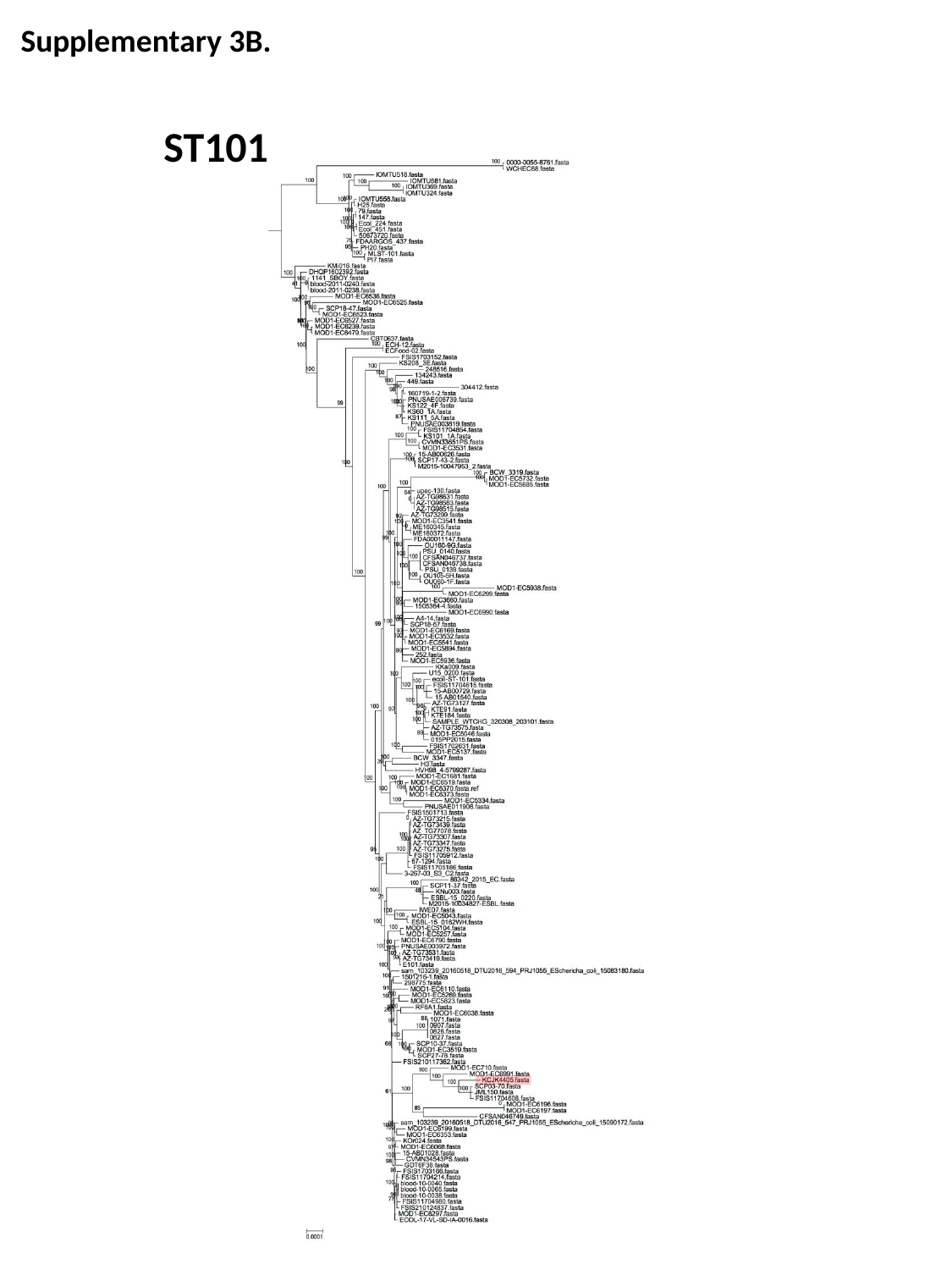

Supplementary 3B.
ST101

## Slide 9
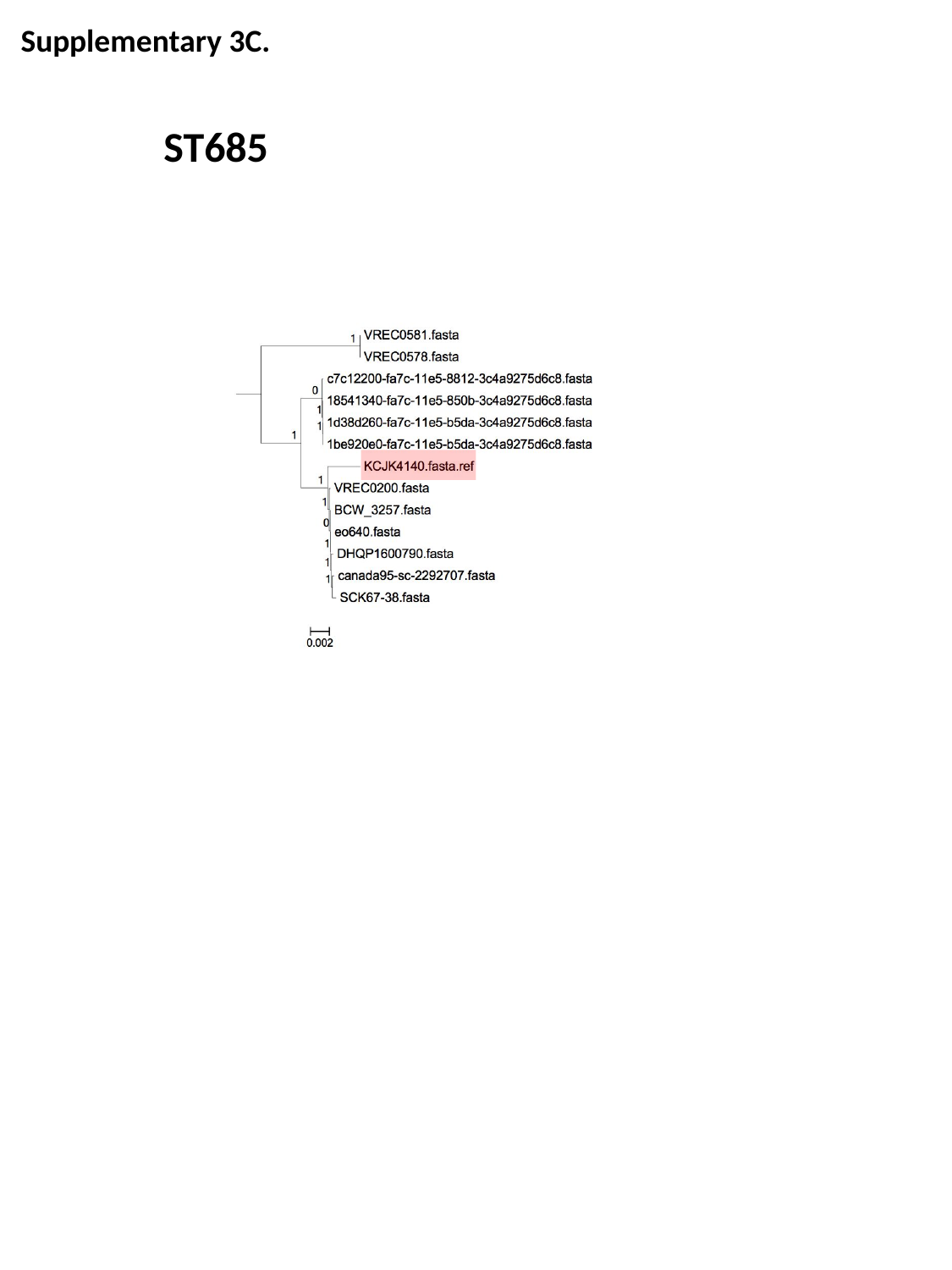

Supplementary 3C.
ST685

## Slide 10
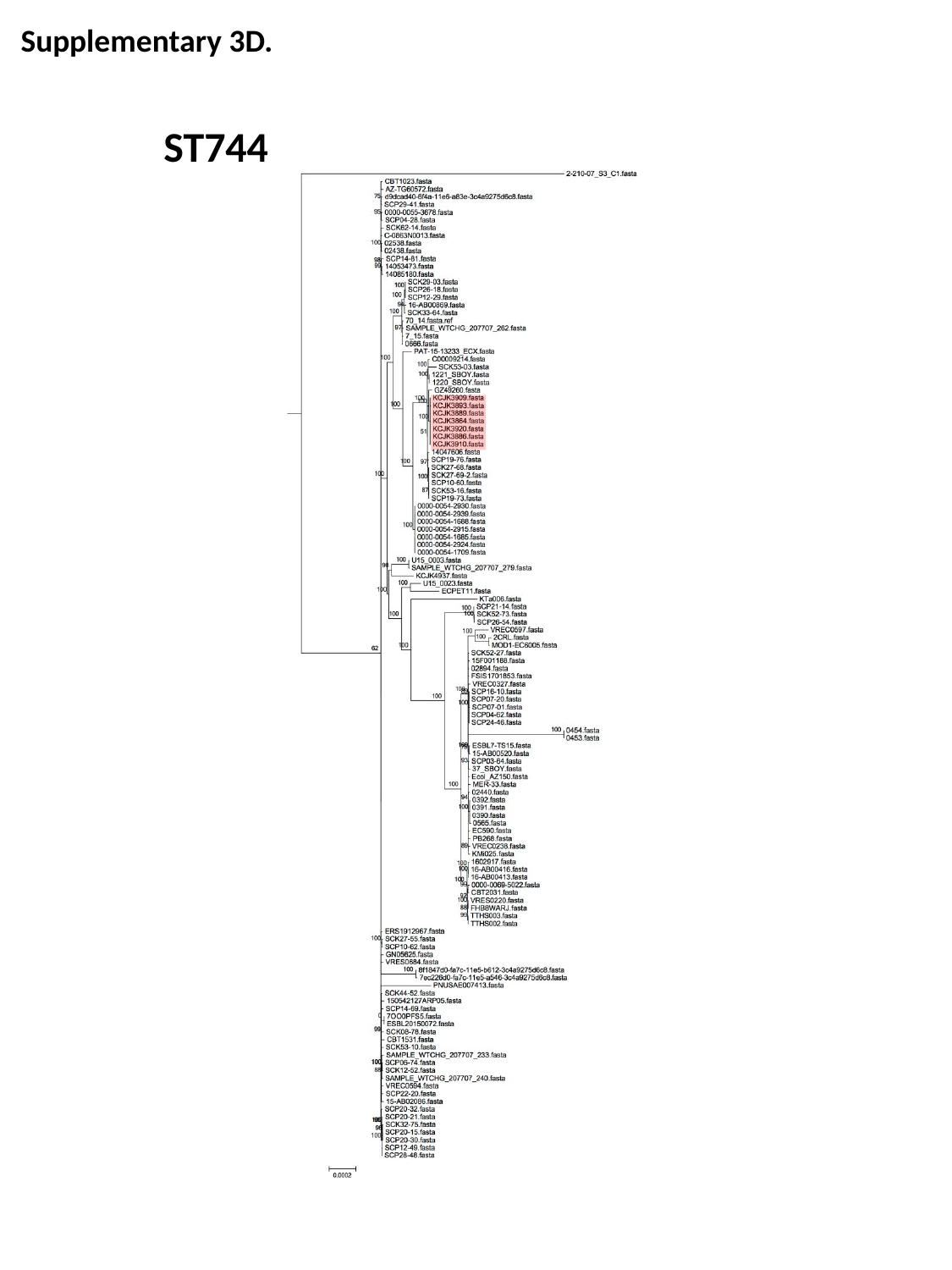

Supplementary 3D.
ST744

## Slide 11
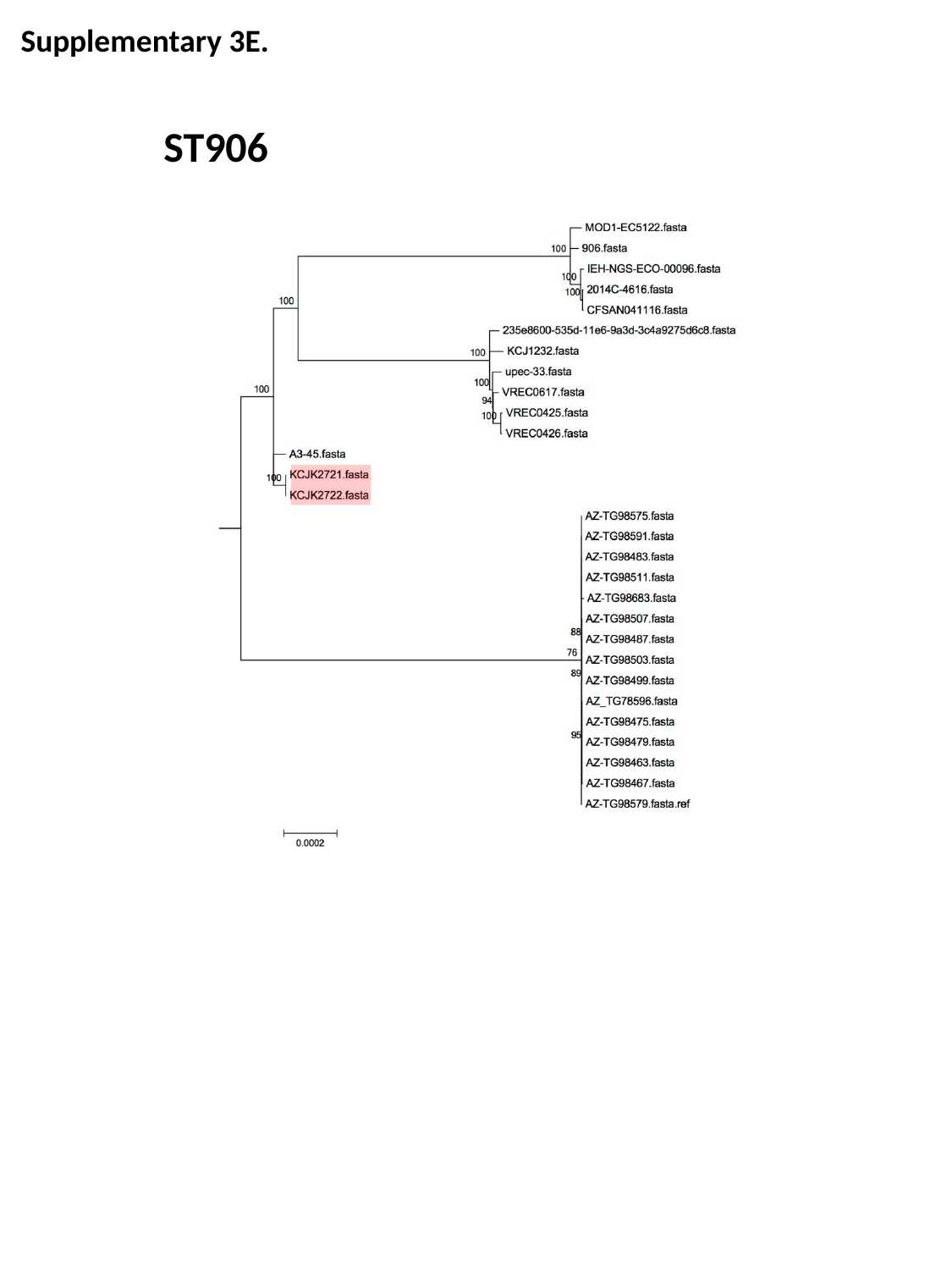

Supplementary 3E.
ST906

## Slide 12
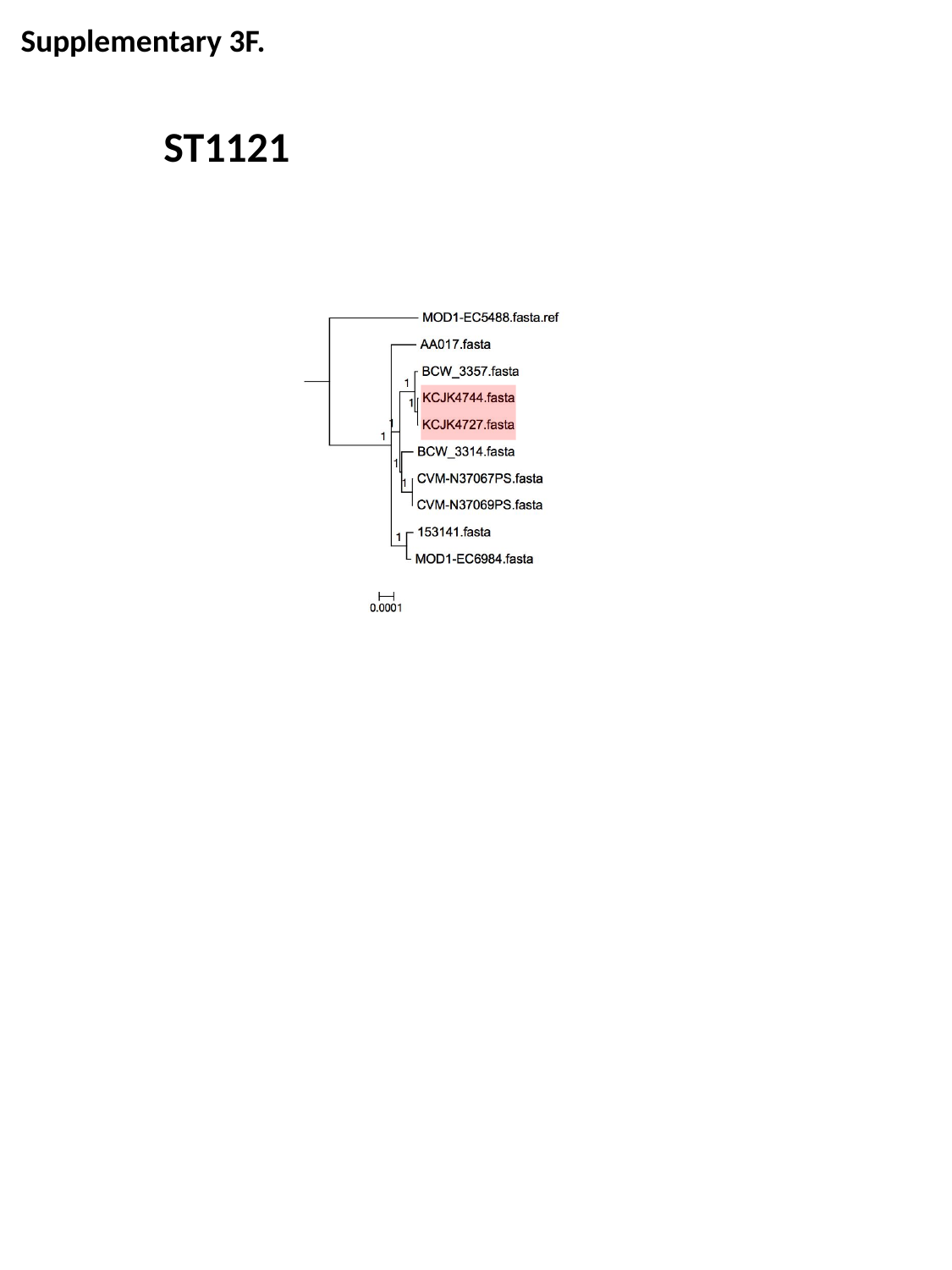

Supplementary 3F.
ST1121

## Slide 13
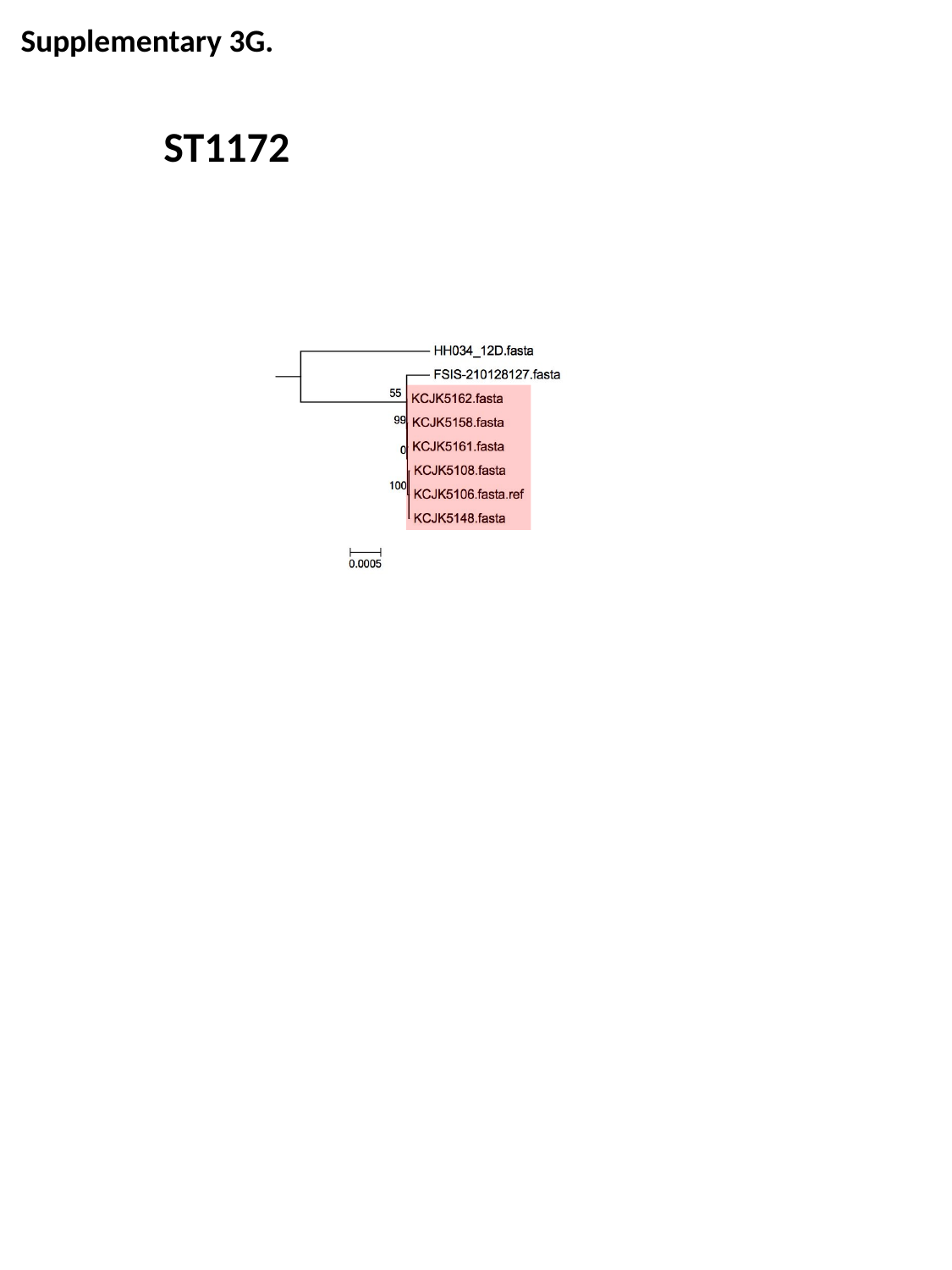

Supplementary 3G.
ST1172

## Slide 14
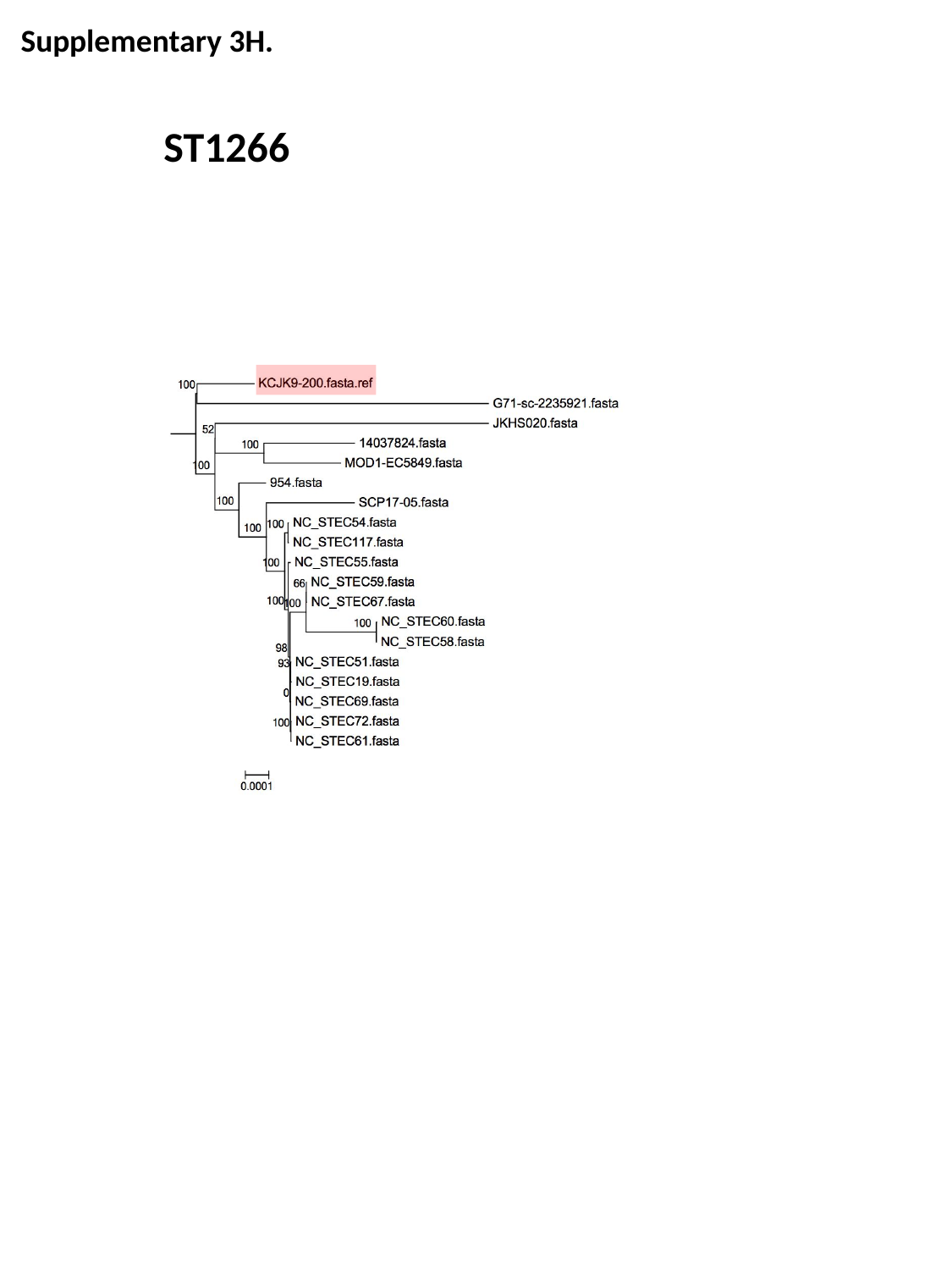

Supplementary 3H.
ST1266

## Slide 15
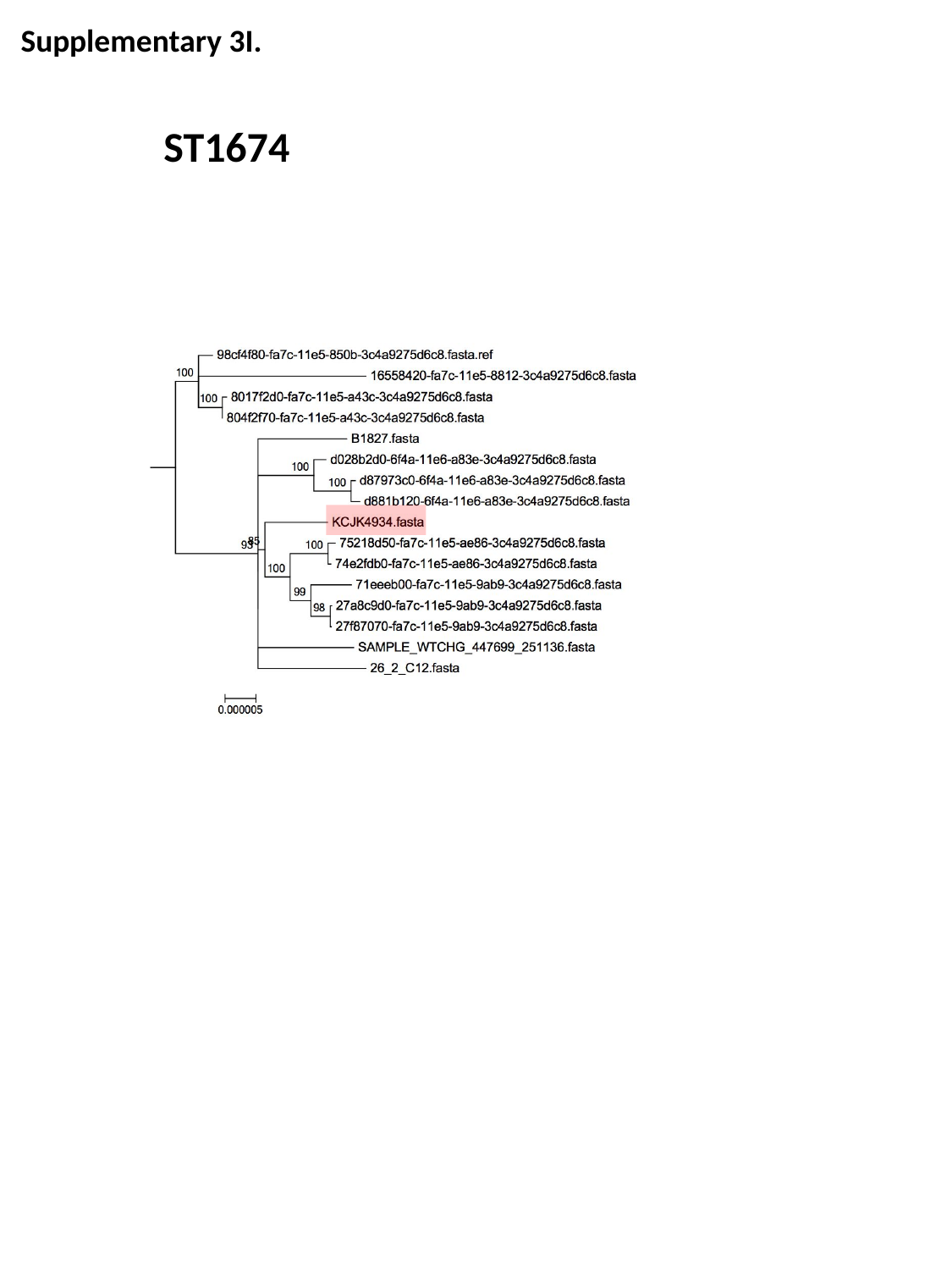

Supplementary 3I.
ST1674

## Slide 16
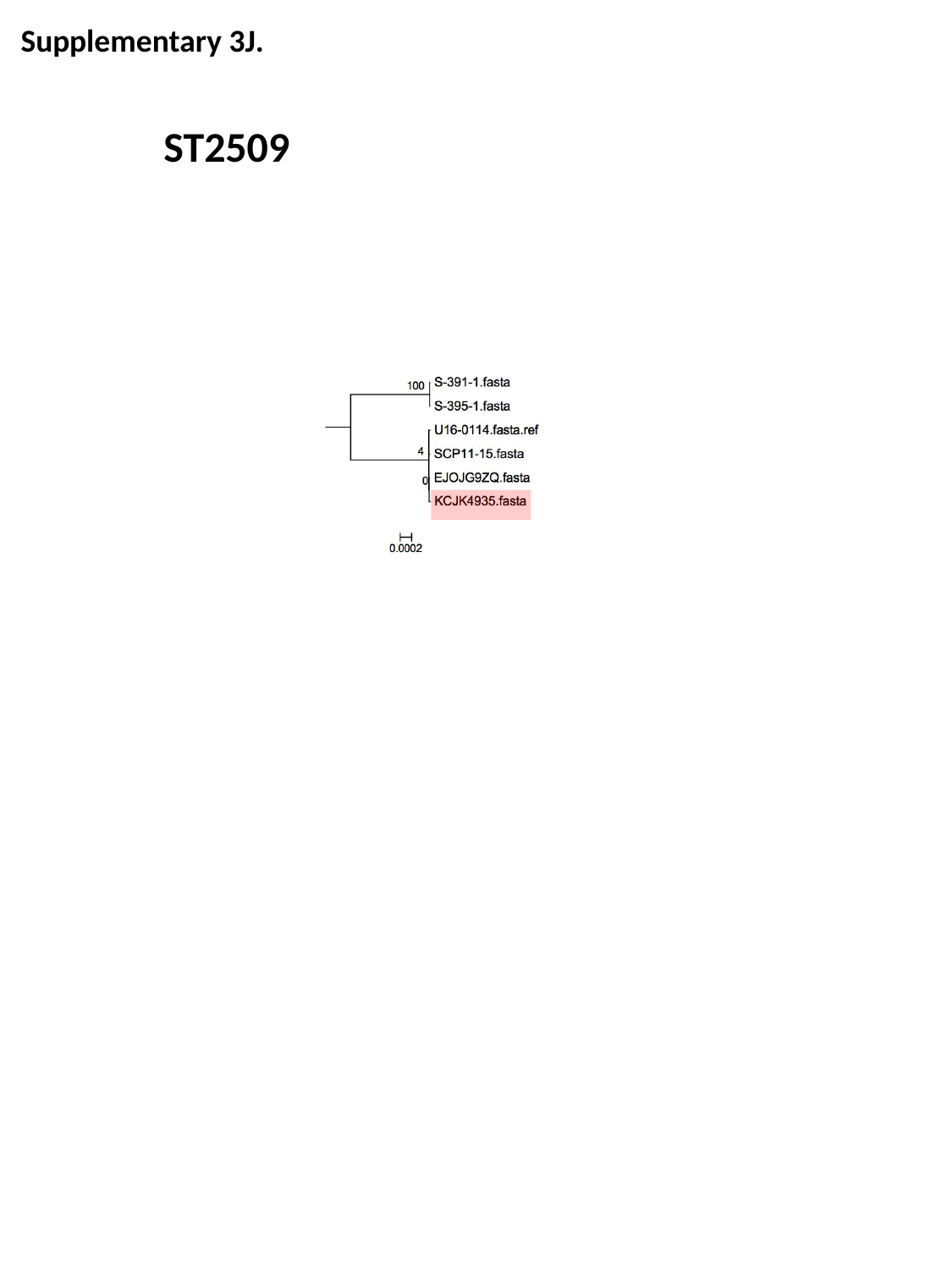

Supplementary 3J.
ST2509

## Slide 17
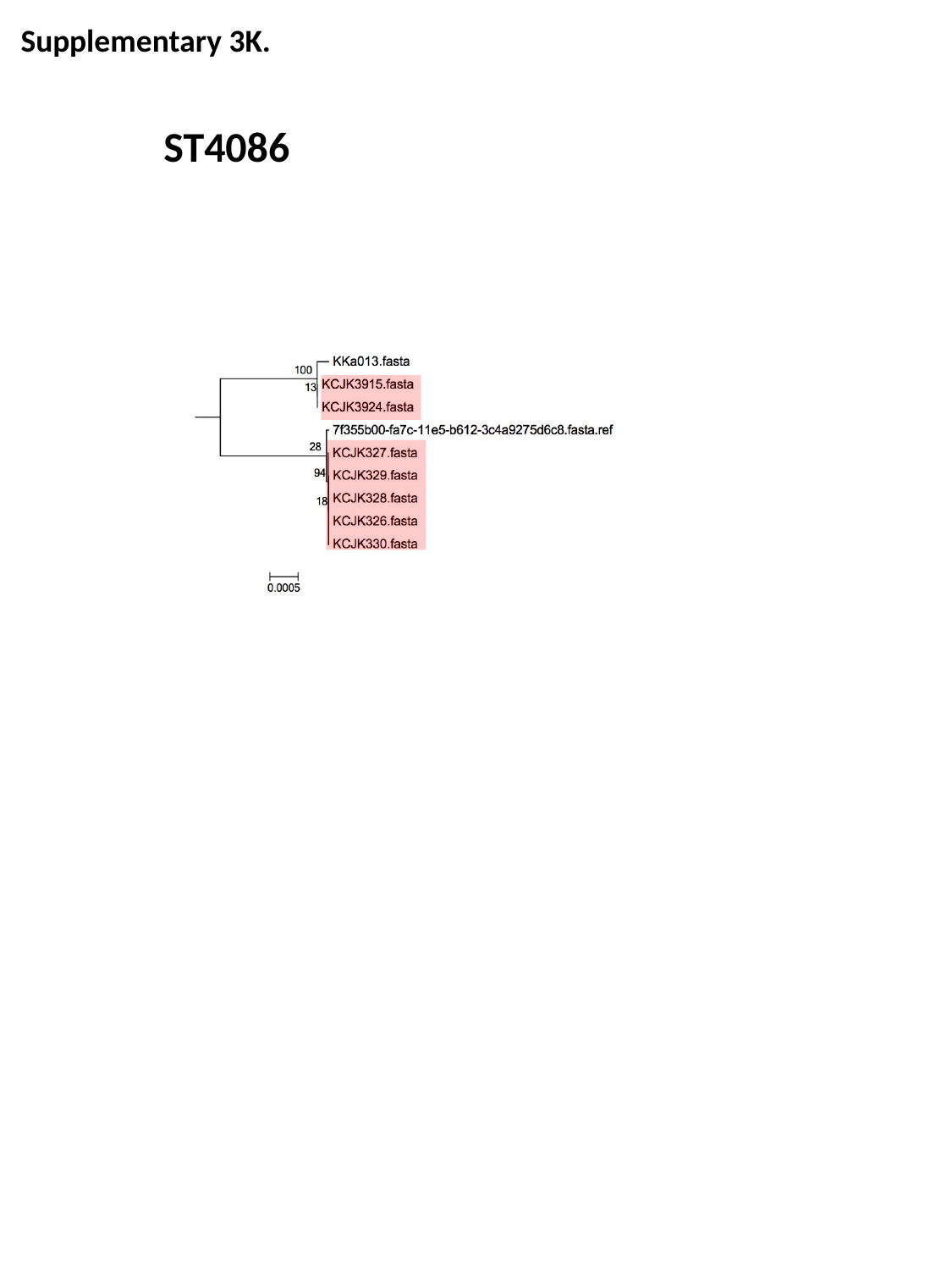

Supplementary 3K.
ST4086

## Slide 18
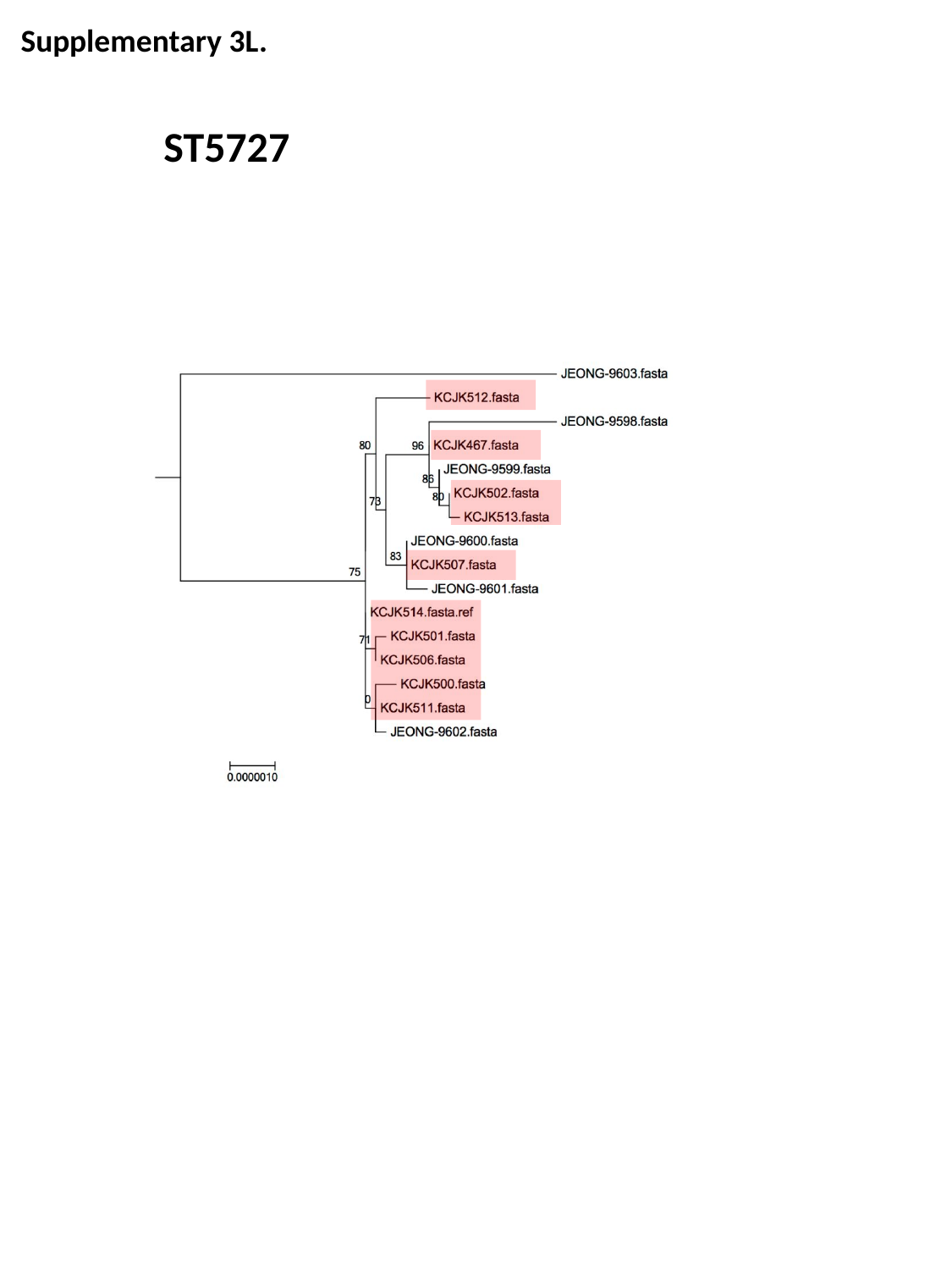

Supplementary 3L.
ST5727

## Slide 19
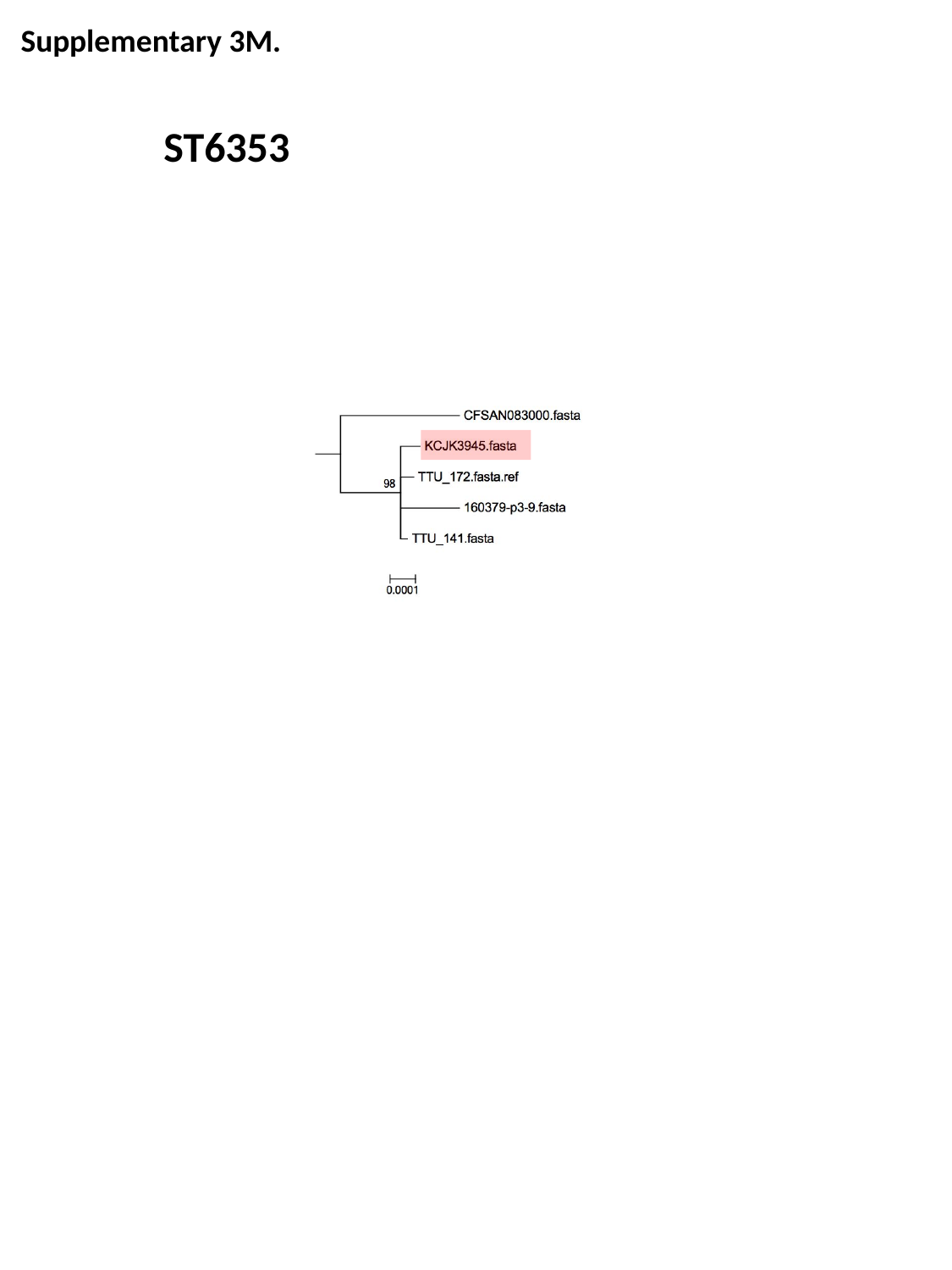

Supplementary 3M.
ST6353

## Slide 20
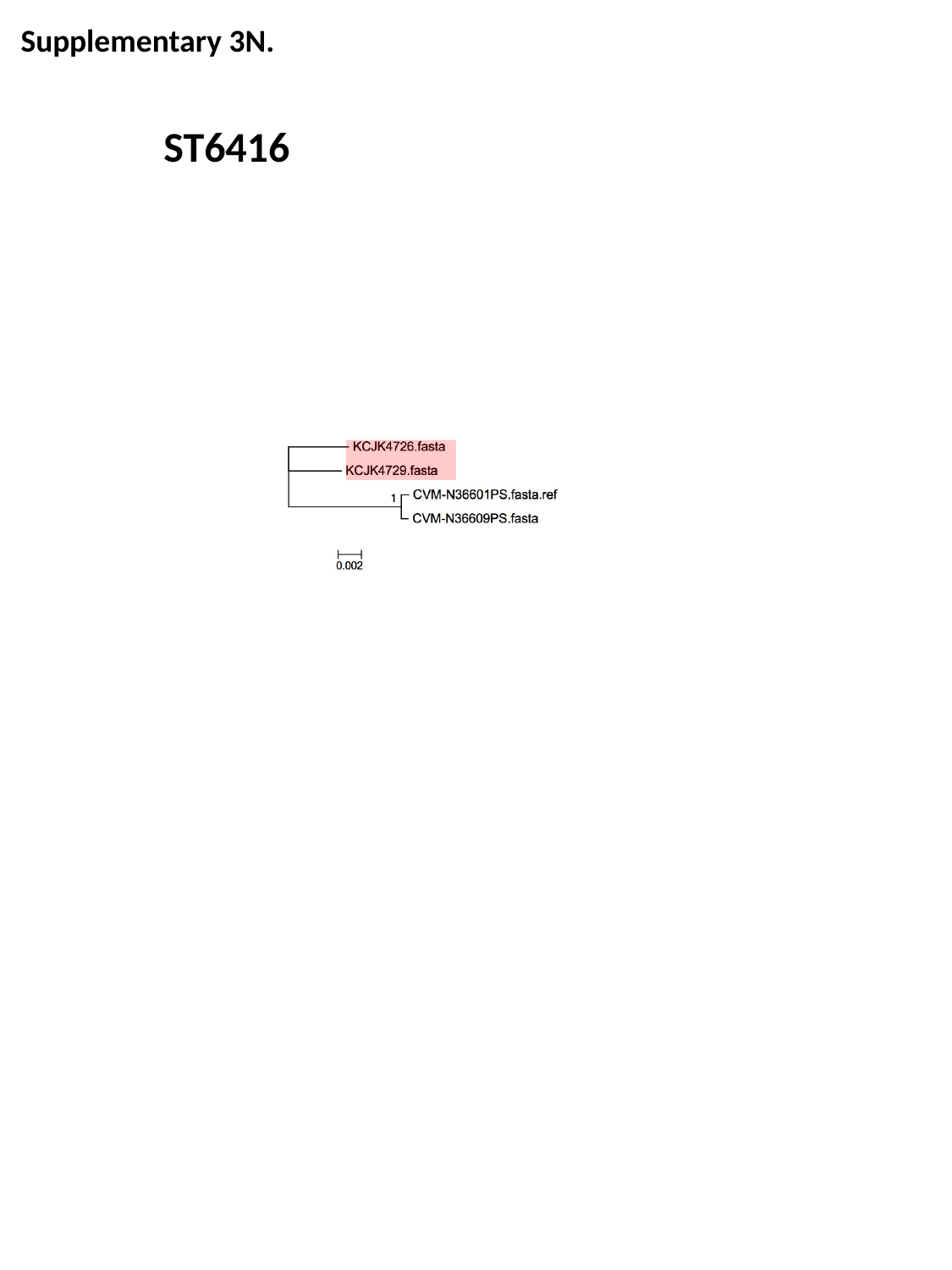

Supplementary 3N.
ST6416

## Slide 21
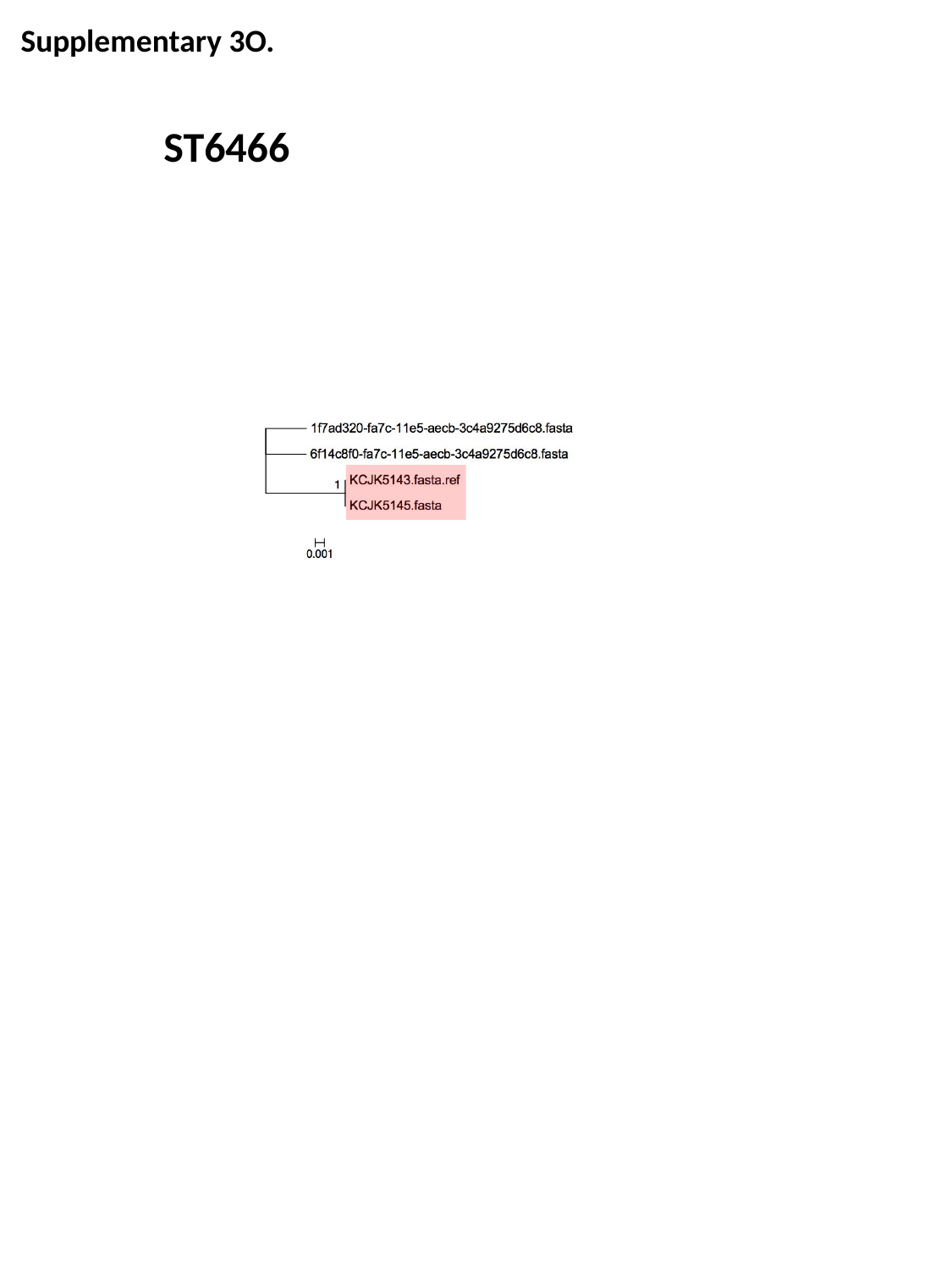

Supplementary 3O.
ST6466
